# Reserpine prolongs lifespan but compromises locomotion and heat-stress resilience in *Drosophila melanogaster*

**DOI:** 10.1038/s41514-026-00329-1

**Published:** 2026-01-12

**Authors:** Vaibhav Tiwary, Nares Trakooljul, Shahaf Peleg

**Affiliations:** 1 Energy Metabolism and Epigenetics, Research Institute for Farm Animal Biology (FBN), Dummerstorf, Germany; 2 Physiological Genomics, Research Institute for Farm Animal Biology (FBN), Dummerstorf, Germany

**Keywords:** Drug discovery, Neuroscience, Physiology

## Abstract

Pharmacological modulation of monoaminergic signaling, a process targeted by many therapeutic and recreational drugs via receptors, transporters, degradation enzymes, or reuptake mechanisms, is emerging as a promising aging intervention and as a strategy to treat various maladies. Monoamines (including dopamine, serotonin, and norepinephrine) are central to the regulation of mood, movement, sleep, memory, and systemic physiology. Here, we demonstrate that Reserpine, chronic inhibitor of the vesicular monoamine transporter (VMAT), robustly extends lifespan in *Drosophila melanogaster* in a dose-dependent manner. However, reserpine-treated flies also exhibit reduced locomotor activity and impaired survival under acute heat-stress, indicating a context-dependent trade-off between lifespan extension and stress resilience. Transcriptomic profiling revealed that reserpine induces a transcriptionally repressed, low-energy state characterized by downregulation of metabolic, immune, and stress-response genes in treated aged animals. Notably, under heat-stress, reserpine blunts the induction of canonical protective genes, including heat shock proteins and antioxidant genes, resulting in increased proteotoxic vulnerability. These findings highlight the potential trade-offs of monoaminergic modulation and support further investigation of VMAT inhibitors, monoamine modulators and other hypertension drugs as geroprotective agents.

## Introduction

Aging is a multifactorial biological process that is characterized by the progressive loss of physiological integrity, functional decline and increased vulnerability to stress and disease. It is orchestrated by an intricate interplay of genetic programs, metabolic states, and environmental exposures^1^. These pathways play central roles in nutrient sensing, metabolic regulation, neurotransmission, and stress response, while their manipulation has been shown to extend lifespan and delay age-related decline across multiple model organisms^1–4^.

A substantial body of research has established that both lifespan and health span can be modulated by targeting evolutionarily conserved signaling pathways, notably the insulin/IGF-1, mTOR and AMPK pathways, as well as monoaminergic signaling^1–5^. For example, change in monoamine concentrations due to overactivation of Monoamine oxidases (MAO) has been found to play a determining role in aging associated pathologies like neurodegeneration, pulmonary diseases, cancer and metabolic disorders including obesity and diabetes^6^. The discoveries of modulating signaling pathways for longevity has prompted efforts to identify pharmacological agents capable of safely mimicking the benefits of dietary restriction and stress resistance without requiring drastic lifestyle interventions^7,8^, including repurposing existing FDA approved drugs for anti-aging therapies^9,10^.

Notably, several clinically approved antihypertensive drugs that were initially developed for cardiovascular control have emerged as candidate geroprotectors. These include, rilmenidine (an I₁-imidazoline receptor agonist), metolazone (a thiazide-like diuretic) and reserpine (a vesicular monoamine transporter inhibitor). In *Caenorhabditis elegans*, each of these three drugs has been shown to extend lifespan and enhance markers of health span, such as locomotion and thermotolerance^11–13^. The convergence of pharmacological action between these antihypertensive compounds and well-characterized longevity interventions underscores the central role of neuroendocrine and metabolic adaptation in aging. However, while promising, these mechanisms remain incompletely understood, with respect to how they affect acute stress resilience, their secondary effects and how and if they can be translated to more complex organisms.

Recent studies in older humans show that long-term use of antihypertensive drugs, especially calcium channel blockers, is linked to slower biological aging and lower frailty. The study by Tang et al.^14^ found that antihypertensive drug use overall was associated with slower biological aging, specifically showing a decrease in DNA methylation age as measured by the PCGrimAge clock. When examining drug subcategories, calcium channel blockers (CCBs) consistently showed links to decreased DNA-methylation ages (PCHorvathAge; PCSkin&bloodAge; PCPhenoAge; PCGrimAge) and in functional biological ages (functional age index; frailty index)^14^. This study population primarily consisted of individuals using medications for conditions such as hypertension, diabetes, and hyperlipidemia, indicating that they had the relevant disorders for which these drugs are prescribed. While antihypertensives clearly reduce cardiovascular risks and mortality, they do not always lead to slower epigenetic aging (DNAmAge), indicating that the relationship between these drugs and biological aging is complex and needs further investigation to understand the mechanisms involved^15^.

Reserpine, a root extract of the perennial shrub of the Rauwolfia family was historically used in traditional medicine and later incorporated into modern pharmacology. More recently it was used as a therapy for reducing blood pressure but nowadays used as a second-line treatment^13,16^. Reserpine is a VMAT-1 and VMAT-2 (vesicular monoamine transporter) inhibitor previously used as an antihypertensive and antipsychotic. By blocking VMAT in neurons, dopamine (DA), serotonin (5-HT) and octopamine are depleted from vesicles, thus reducing their release to the synapse and further to the post-synaptic neuron^17^. VMAT blockade consequently mimics neurotransmitter-deficient states: for example, reserpine-treated cells accumulate cytosolic DA, generate reactive oxygen species (ROS), and trigger apoptosis^18^. Notably, reserpine’s broad action differs from more selective monoamine drugs: for instance, the antipsychotic haloperidol blocks D2 receptors^19^, whereas reserpine’s inhibition of VMAT results in elimination of multiple neurotransmitter stores such as depleting dopamine, serotonin, norepinephrine and histamine^20^. As such, reserpine treatment might result in engagement of complex downstream pathways which could be resulting in extended lifespan.

In the early 1950s reserpine was introduced as a first‑line antihypertensive and, for a short time, as an antipsychotic^20^. Clinicians observed that reserpine often induced depression-like symptoms that typically abated after stopping the drug. These findings underpinned the mono‑amine‑depletion hypothesis, which posits that loss of catecholamines (dopamine, norepinephrine) causes depression and helped to justify the drug’s withdrawal from routine use^20^. A 2023 systematic review Strawbridge et al.^20^ examined 35 human studies on reserpine and concluded that the link between mono‑amine depletion and depression is inconsistent and poorly supported, urging a nuanced reinterpretation of the classic monoamine hypothesis rather than a simple causation model. Another pilot study by Siddiqui et al.^21^ suggests that reserpine could also be reconsidered for refractory hypertension^22^.

Reserpine has been shown to extend lifespan in *Caenorhabditis elegans* through two distinct and parallel pathways^3^. One pathway involves dopaminergic signaling via the D2-like receptor DOP-3, which acts through the G-protein GOA-1 to activate the transcription factor JUN-1, ultimately upregulating the ABC transporter MRP-1; a gene associated with detoxification and stress resistance. The second pathway involves ERI-1, a 3′-5′ exoribonuclease that suppresses RNA interference; its loss enhances neuronal sensitivity to RNAi and is also required for the lifespan-extending effects of reserpine. These mechanisms operate independently of canonical longevity pathways such as insulin/IGF-1 signaling or dietary restriction in *C. elegans*^3^. Many long-lived worm mutants and flies exhibiting longevity using compounds exhibit better stress tolerance including heat-stress tolerance^23–26^. In addition, in *Caenorhabditis elegans*, reserpine confers a ~40% increase in survival at 35 °C, paralleling the stress resistance of established longevity mutants^13^.

While this study provides key mechanistic insights into how reserpine promotes longevity in *C. elegans*, it does not offer a broader systems-level view of how these pathways integrate and modulate with metabolic, immune, or stress-response networks, nor does it address whether reserpine can operate in more complex organisms for lifespan extension.

Notably, a key question in translational Geroscience is whether interventions that promote lifespan necessarily enhance stress resistance or whether trade-offs emerge between longevity and stress adaptability^27,28^. For example, our recent work in flies revealed that reducing the levels of chm, a lysine acetyltransferase, leads to lifespan extension in flies while impairing their capacity to survive starvation in cold temperatures^29^. To bridge this gap, we employed the model organism *Drosophila melanogaster* to investigate the effects of chronic dietary reserpine supplementation. Many of the neurotransmitters utilized by mammals are also used by flies, such as the monoamines histamine, serotonin (5HT), and dopamine (DA)^30^. Since histamine is produced by the enzyme histidine decarboxylase and plays a part in the visual system, it has been thoroughly studied in insects^31^. In *Drosophila*, octopamine is a major stress/arousal transmitter and analog of norepinephrine^32^ while dopamine and serotonin regulate arousal, feeding and mood^33–35^. Conserved VMAT function underlies reserpine’s pharmacology in *Drosophila*. *Drosophila* VMAT (dVMAT) shares conserved structure and function with mammalian VMAT2, as demonstrated by genetic and pharmacological evidence. The primary structure of dVMAT and mammalian VMATs are similar throughout the 12 predicted transmembrane domains, the regions likely to be responsible for substrate recognition and transport^36^ dVMAT is also inhibited by the reserpine at sub-micromolar concentrations^36^. Indeed, neurotransmitter substrate-specificity and relative affinity of dVMAT are generally similar to mammalian VMATs; Nall and Sehgal^37^ showed that VMAT-null mutants are resistant to reserpine’s behavioral effects, confirming dVMAT as the principal in vivo target^37^. In contrast to the mammalian genome which contains two distinct VMAT genes while flies contains only one. Another study by Chang et al.^38^ shows overexpression of isoform of *Drosophila* VMAT (DVMAT-A) potentiates grooming and locomotion behavior and is reversed by inhibition of VMAT by reserpine showing the role of both VMAT and Reserpine in locomotion and its potential in studying neuropsychiatric illnesses^38^. Collectively, these studies establish that reserpine’s mechanism of action in flies is conserved at the molecular and functional levels with mammalian VMAT2. Overall, *Drosophila* possesses a conserved neuroendocrine architecture^30^, robust stress-response pathways, and well-characterized aging phenotypes^39^, making it an ideal system to explore pharmacological interventions^40^ with potential relevance to human biology^41^.

In this study, we assessed how reserpine affects lifespan, locomotor function, heat-stress resilience, and global gene expression patterns. By integrating survival assays and transcriptomic profiling, our study uncovers how pharmacological modulation of monoaminergic signaling induces a low-energy, longevity-associated state but at the cost of reduced locomotor activity and suppressed acute stress resilience such as heat-stress. These results reveal a mechanistic trade-off with implications for both fundamental aging biology and the rational development of geroprotective therapies using FDA-approved drugs.

## Results

### Lifespan extension by chronic dietary reserpine treatment in male *Drosophila*

We first tested the impact of various concentrations of reserpine on the lifespan of male flies. The first pilot experiment was with various concentrations ranging from 0 to 800 µM of drug in fly food (Fig. S1). We noticed a detectable lifespan increase in reserpine-treated flies between 200 µM to 800 µM. We next increased the concentration of the drug to 1000 µM along with a condition of start of drug treatment at around half the lifespan of fly with (0–800 µM; reserpine introduced at day 31) as 800 µM was most promising in the first pilot. Starting the treatment in midlife with 800 µM failed to increase the median lifespan, thus suggesting that early life treatment was crucial for lifespan extension. Furthermore, increasing the treatment to 1000 µM showed further increase lifespan compared with 800 µM (Fig. S2).

**Fig. 1:**
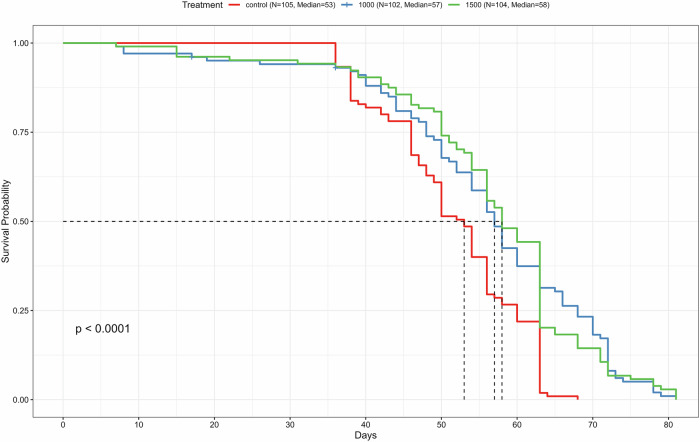
Reserpine treatment extends lifespan in male *Drosophila melanogaster*. Kaplan–Meier survival curves for male flies chronically treated with 0 (control), 1000, or 1500 µM reserpine under standard conditions. Median lifespans were 53 days for control (*N* = 105), 57 days for 1000 µM (*N* = 102), and 58 days for 1500 µM reserpine-treated flies (*N* = 104). Each treatment group included 3 independent biological replicates each containing an average of *n* ~ 35 flies per vial. Overall group differences were statistically significant (log-rank test, *p* < 0.0001).

We next focused on higher concentrations of 1000 µM and 1500 µM. Indeed, chronic dietary administration of higher dosage reserpine significantly extended the median, mean, and maximum lifespan (Fig. 1). Specifically, Kaplan–Meier survival analysis demonstrated an increase in median survival from 53 days in controls to 57 days at 1000 µM and 58 days at 1500 µM reserpine (log-rank test: *p* < 0.0001 for all comparisons). The mean lifespan also increased from 51.5 days in controls to 54.9 days at 1000 µM and 56.7 days at 1500 µM. The maximum lifespan rose from 68 days in controls to 81 days at both 1000 µM and 1500 µM concentrations (Table 1).

**Table 1:** Summary of median, mean and maximum lifespan extension under Reserpine treatment

	N	Median lifespan (days)	Mean lifespan (days)	Max lifespan (days)
Control	105	53	51.5	68
1000 µM	102	57	54.9	81
1500 µM	104	58	56.7	81

### Reserpine reduces climbing ability and heat-stress tolerance

**Fig. 2:**
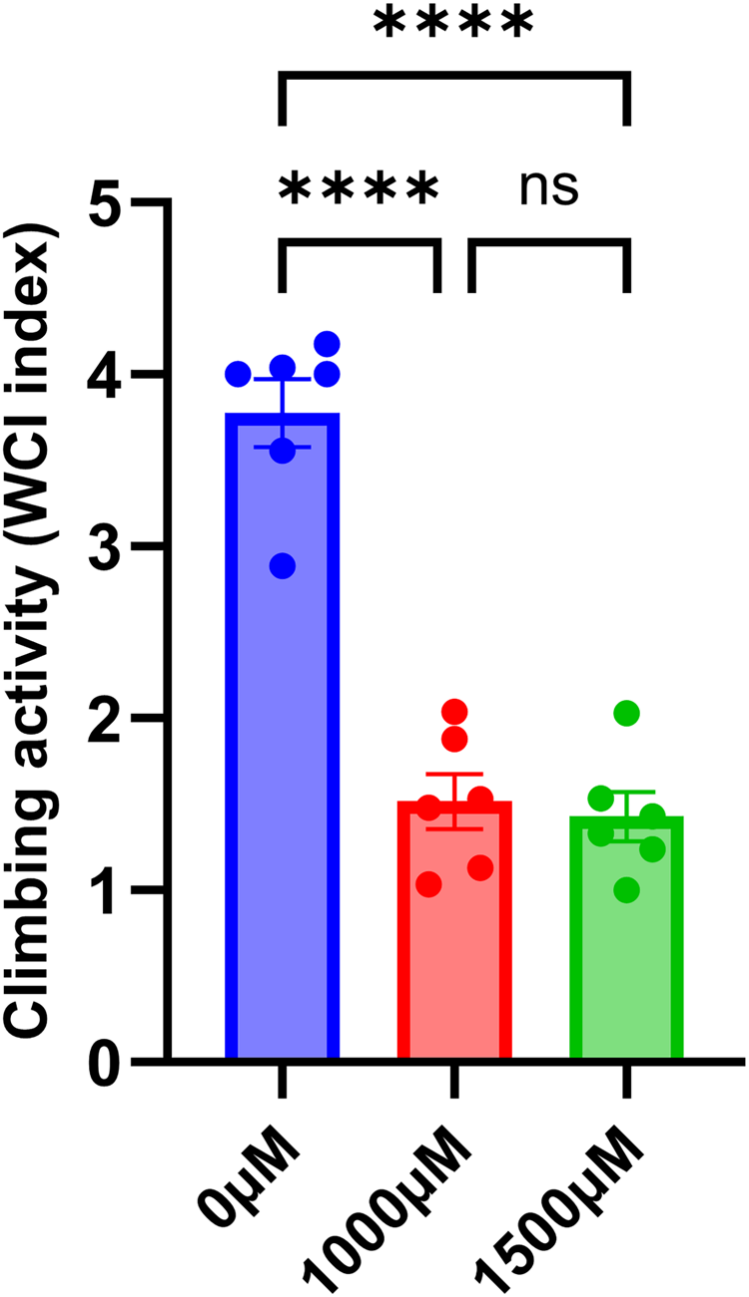
Chronic Reserpine treatment impairs climbing ability in adult *Drosophila*. Flies were treated with 0 µM, 1000 µM, or 1500 µM of the test compound for 12 days, and climbing ability was assessed on day 13 using a negative geotaxis assay. Each bar represents the Weighted Climbing Index (WCI), calculated based on fly distribution across five equal vertical quadrants at 10 s post-stimulation. Each point represents one biological replicate (*n* = 6 per group; 25–31 flies per replicate). Bars show mean ± SEM. Statistical analysis was performed using one-way ANOVA with Tukey’s multiple comparisons test. ****p < 0.0001; ns not significant.

Previous studies in *C. elegans* showed reserpine increased thermotolerance and locomotion^13^. Contrarily, it was evident that reserpine treated flies exhibit decreased activity while performing survival assays. To quantify the fly activity, we conducted climbing assay^42^. Reserpine-treated flies had severely reduced activity in climbing assay after 13 days of treatment. Tukey’s post hoc comparisons revealed a significant reduction in Weighted Climbing Index (WCI) in both the 1000 µM (mean = 1.52 ± 0.16) and 1500 µM (mean = 1.43 ± 0.14) groups compared to the control group (mean = 3.78 ± 0.20). No significant difference was observed between the 1000 µM and 1500 µM groups (*p* = 0.9286). These results indicate that chronic treatment with the drug at both concentrations significantly impairs climbing ability in adult flies, with no additional impairment at the higher dose (Fig. 2).

**Fig. 3:**
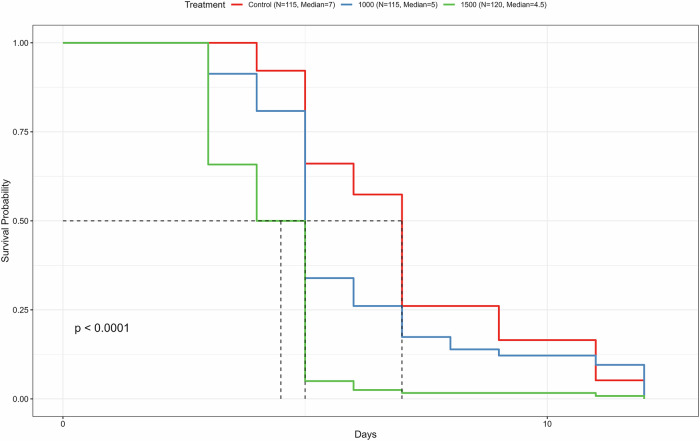
Reserpine treatment increases mortality under heat-stress in young male flies. Kaplan–Meier survival analysis under continuous heat-stress (31 °C) shows median survival times of 7 days (control), 5 days (1000 µM), and 4.5 days (1500 µM). Each treatment group included 3 independent biological replicates each containing an average of *n* ~ 35 flies per vial. Overall group differences were significant (log-rank test, *p* < 0.0001). Day ‘0’ marks the first day after 12 days at 25 °C.

Thermal stress is a key ecological constraint shaping survival and distribution in *Drosophila*, and standardized heat-tolerance assays provide sensitive, complementary readouts of physiological resilience^43^. In *Drosophila melanogaster*, pharmacological dVMAT inhibition impairs heat-avoidance behavior and reduces thermotolerance, linking monoaminergic depletion to stress susceptibility^44^ conversely Srivastava et al.^13^ showed increased heat-stress tolerance in worms upon reserpine treatment. Monoaminergic signaling modulates stress-related behaviors and physiology^45–47^; thus, VMAT inhibition by reserpine was hypothesized to impair heat tolerance while altering longevity-associated pathways. Therefore, we subjected young control and treated flies to dry heat-stress after 12 days of treatment at standard conditions (see methods). Under continuous heat-stress conditions starting at day 12 of their life (31 °C), reserpine significantly reduced survival in flies in a dose-dependent manner (Fig. 3). Median survival decreased from 7 days in the control group (*n* = 115) to 5 days with 1000 µM reserpine (*n* = 115), and 4.5 days with 1500 µM reserpine (*n* = 120) from the start of the heat stress. Correspondingly, the mean lifespan declined from 7.06 days in controls to 5.97 days and 4.31 days in the 1000 µM and 1500 µM groups, respectively, while the maximum lifespan under the heat-stress remained at 12 days across all groups. Log-rank analysis confirmed a highly significant difference between treatment groups (*p* < 0.0001). These results are consistent with study by Bressan et al.^44^ who found that reserpine impaired thermal tolerance, heat-avoidance behavior, and locomotor activity in *Drosophila*^44^. Together, the data suggest that while reserpine extends lifespan under normal conditions, it compromises the organism’s ability to withstand acute environmental stress and reduces activity.

### Reserpine alters the transcriptome of aged *Drosophila*

**Fig. 4:**
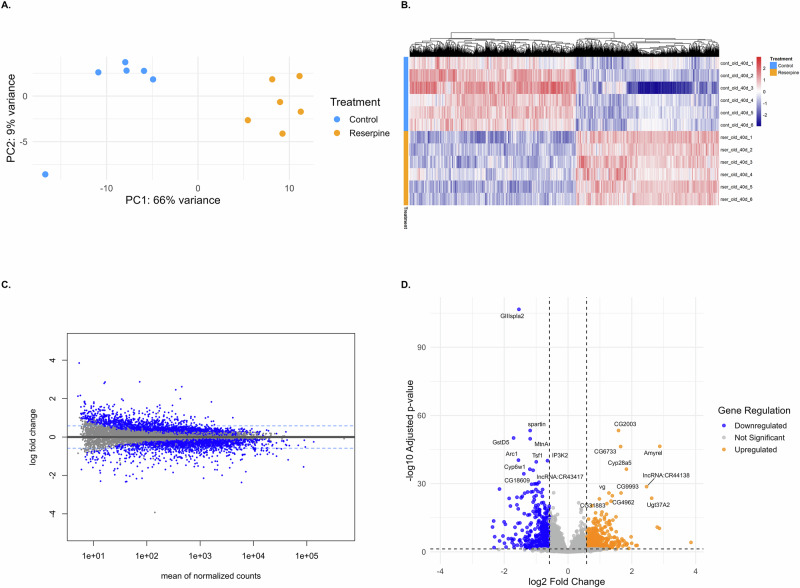
Transcriptomic profiling of aged flies (day 43) treated with reserpine compared to controls. **A** PCA plot showing transcriptome-wide differences between old control (red) and reserpine-treated old flies (blue). PC1 explains 66% of the total variance, showing a clear separation based on treatment. **B** Heatmap of DEGs (adjusted *p*-value < 0.05) with hierarchical clustering, displaying gene expression patterns across groups. **C** MA plot displaying log2 fold change versus mean normalized counts. Genes with |log2FC| > 0.58 and padj < 0.05 are marked in blue. Horizontal dashed lines represent ±0.58 thresholds. **D** Volcano plot of DEGs highlighting significantly upregulated (red) and downregulated (blue) genes, with the top 10 genes labeled by gene symbol and ranked by padj value.

Various studies have shown that there are distinct and significant transcriptomic changes and metabolic shifts that occurs during aging in fruit flies^48,49^. In addition, Heat shock proteins (Hsps) are known modulators of lifespan and stress resistance in *Drosophila*^50^. Therefore, we first compared the transcriptome of 43 days control vs chronic reserpine treated male flies. RNA-seq analyses revealed that reserpine induces a robust, condition-specific transcriptomic reprogramming in aged *Drosophila* (Fig. 4). Principal component analysis (PCA) demonstrated a clear separation between control and reserpine-treated samples, highlighting a substantial impact of reserpine on the aged transcriptome (Fig. 4A). A heatmap of normalized expression values for differentially expressed genes (DEGs; padj < 0.05, *n* = 3964) showed distinct expression patterns and consistent clustering by treatment group (Fig. 4B). MA plot highlights significantly changed genes (adjusted *p* < 0.05) with log2 fold change ±0.58. Many genes showed moderate yet significant fold changes, reflecting a subtle but coordinated transcriptomic reprogramming in response to reserpine (Fig. 4C). The volcano plot summarizes statistical significance versus fold change. Genes such as *Gillspa92*, *spn1*, *GstD5*, *Cyp6g1*, and *lncRNA:CR43417* were significantly downregulated, whereas *Cyp28a5*, *Cyp6w1*, *CG2003*, and *Amyrel* were significantly upregulated (Fig. 4D).

### Reserpine metabolic reprogramming and stress-defense suppression in old flies

**Fig. 5:**
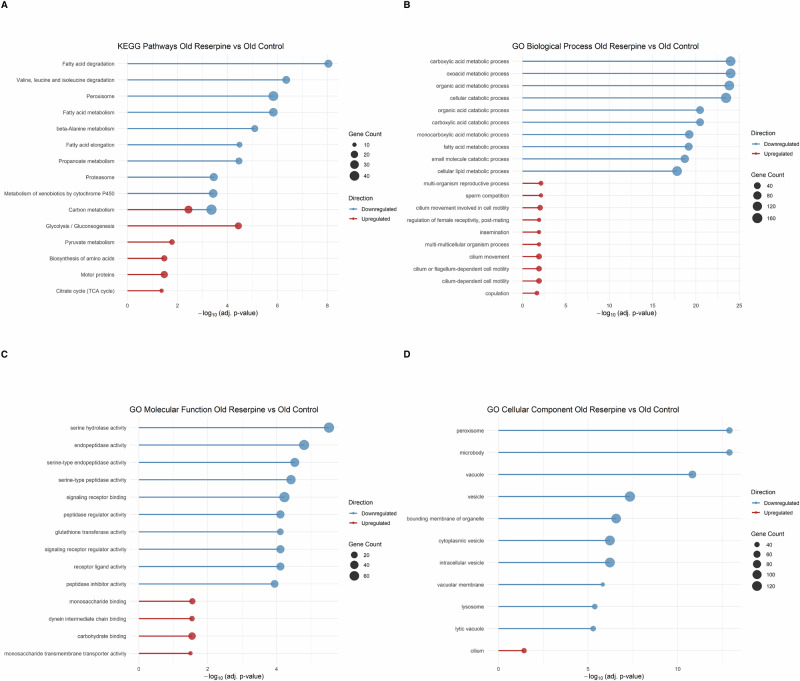
Over-representation analysis (ORA) of transcriptomic changes in reserpine-treated aged flies (Reserpine Old vs. Control Old). **A** KEGG pathway enrichment; **B** GO Biological Process; **C** GO Molecular Function; **D** GO Cellular Component. Downregulated pathways include fatty acid degradation, proteasome, peroxisome, and detoxification-related terms, while upregulated pathways highlight glycolysis, TCA cycle, and cilium-related processes. Analyses were performed using clusterProfiler. Each Dot size indicates the number of genes enriched per term, and position on the x-axis represents −log₁₀ (adjusted *p*-value).

Over-Representation Analysis (ORA) of Gene Ontology (GO) terms including Biological Process (BP), Molecular Function (MF), and Cellular Component (CC) and KEGG pathways (Fig. 5A–D), along with Gene Set Enrichment Analysis (GSEA) of the same categories (Fig. S3A–D), revealed a strong downregulation of gene sets associated with fatty acid metabolism, inflammation, catabolism, proteostasis, peroxisomal and mitochondrial function and cytochrome P450-mediated detoxification. In parallel, reserpine treatment weakly upregulated pathways related to glycolysis, pyruvate metabolism and the TCA cycle (Fig. 5A), highlighting a metabolic reprogramming of glycolytic and mitochondrial energy metabolism. Together, these enrichment patterns indicate a transcriptional state that reshapes core bioenergetic processes while mitigating age-related metabolic and proteolytic stress. As seen in KEGG enrichment, carbon metabolism is both highly downregulated with greater gene count and significance in treated vs control flies, while parts of carbon metabolism are also relatively weakly upregulated further indicating a complex carbon metabolism transcriptional shift or reprogramming (Figs. 5A, B, S3A, B). This pattern suggests a form of mitochondrial remodeling in aged flies, favoring metabolic flexibility and reduced redox stress. Such shifts have been observed in other models of extended lifespan, including dietary restriction (DR), Indy mutants that mimic DR, and PRC2-deficient flies^51–54^. A notable finding was the strong enrichment of serine hydrolase-related activity and serine type endopeptidase activity among the top four downregulated Molecular Function (MF) GO terms (Figs. 5B,C, S3B,C). Serine hydrolases include proteases with roles in immune activation, stress response, and proteostasis, such as Persephone, a key immune protease in flies^55,56^. Dysregulated serine metabolism has been implicated in chronic inflammation^57^ and aging-related diseases^57^.

Collectively, these findings suggest that chronic reserpine exposure in aged flies leads to a transcriptomic state marked by downregulation of stress response and metabolic genes, potentially reflecting a shift toward a low-energy expenditure phenotype with implications for aging and longevity as also indicated by reduced activity in climbing assay.

### Reserpine reshapes the heat-stress transcriptome in young flies

**Fig. 6:**
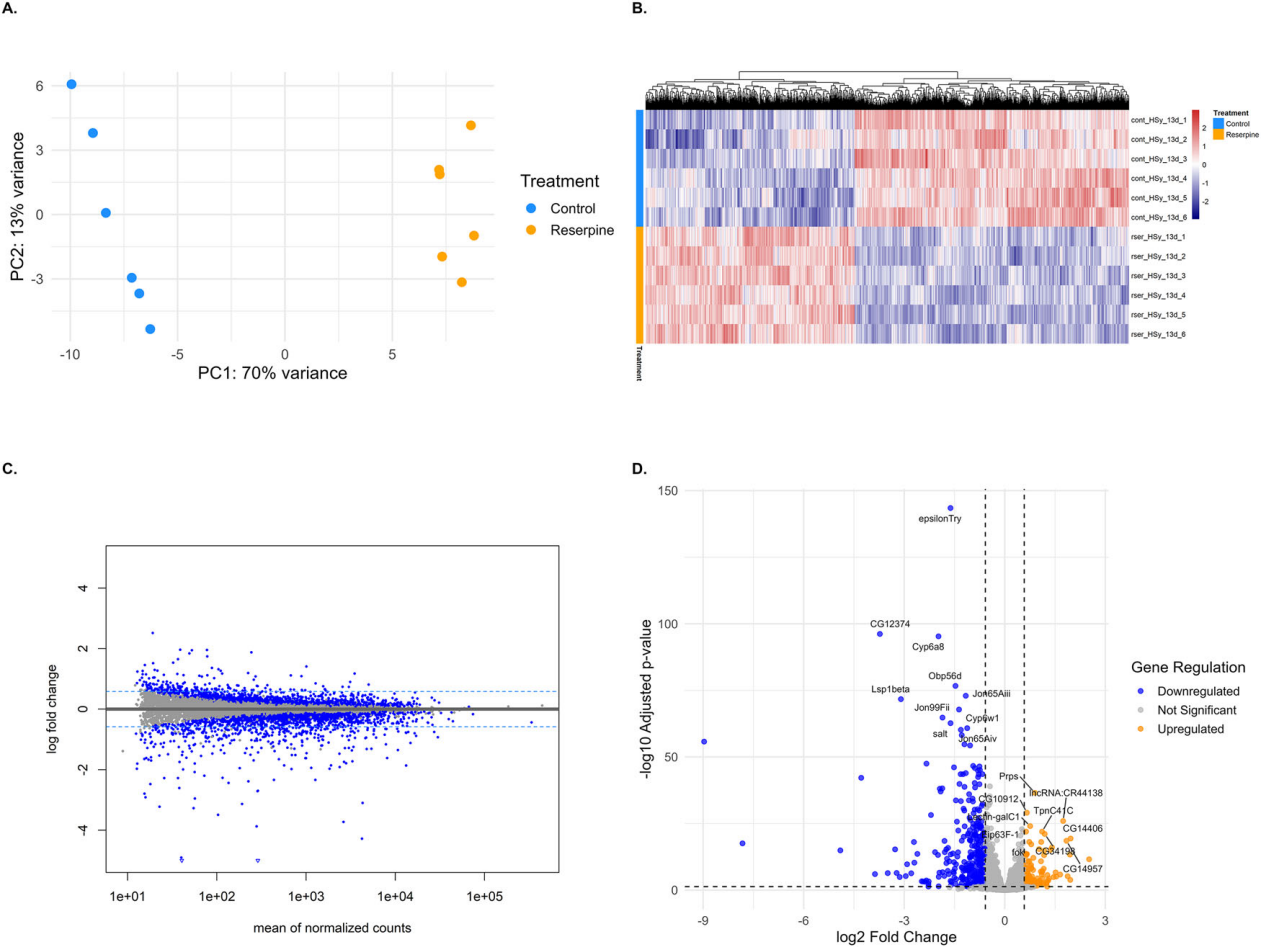
Transcriptomic profiling of heat-stress exposed young flies. **A** Principal component analysis (PCA) showing distinct clustering of samples by treatment, indicating transcriptomic differences induced by reserpine under heat-stress. **B** Heatmap of differentially expressed genes (DEGs; adjusted *p*-value < 0.05), illustrating patterns of gene expression across samples. **C** MA plot depicting the relationship between mean expression and log2 fold changes for all genes, with thresholds indicated for differential expression. **D** Volcano plot illustrating log2 fold change versus −log10 adjusted *p*-values of DEGs. Both significantly upregulated and downregulated genes are highlighted, with the top 10 genes labeled.

Next we compared the transcriptome profiles of 13 days young flies following heat-stressed between control and chronic reserpine treated (12 days standard temperature + 24 h treatment at 31 °C, see methods). PCA revealed strong separation between groups, with PC1 explaining 70% of the variance, highlighting the dominant influence of reserpine in shaping transcriptomic responses to heat-stress (Fig. 6A). Similarly to aged flies, reserpine induced a marked shift in gene expression under heat-stress conditions in young flies. The heatmap of DEGs (padj < 0.05, *n* = 2871) shows distinct clustering between treated and control samples, indicating a consistent reserpine effect on the transcriptome (Fig. 6B). MA plots show again predominant downregulation of significantly altered genes, supporting a suppression of energy-demanding processes under reserpine treatment (Fig. 6C). Volcano plots illustrate significant downregulation of genes involved in digestion (*Jon99Fii*, *Jon65Aiii*), detoxification (*Cyp6a8*) and sensory perception (Obp56d) (Fig. 6D). Additional downregulated genes include *epsilonTry* and *Lsp1beta*, associated with protease activity and storage protein function, suggesting suppression of metabolic and sensory pathways during heat-stress. Upregulated genes included *Prps* and *Lectin-galC1*, linked to biosynthetic and immune responses, and fork, a transcription factor involved in stress and longevity. TpnC41C, a calcium-binding protein, was also upregulated, potentially reflecting muscle-related stress.

### Reserpine impairs heat-stress response and suppresses stress-defense pathways

**Fig. 7:**
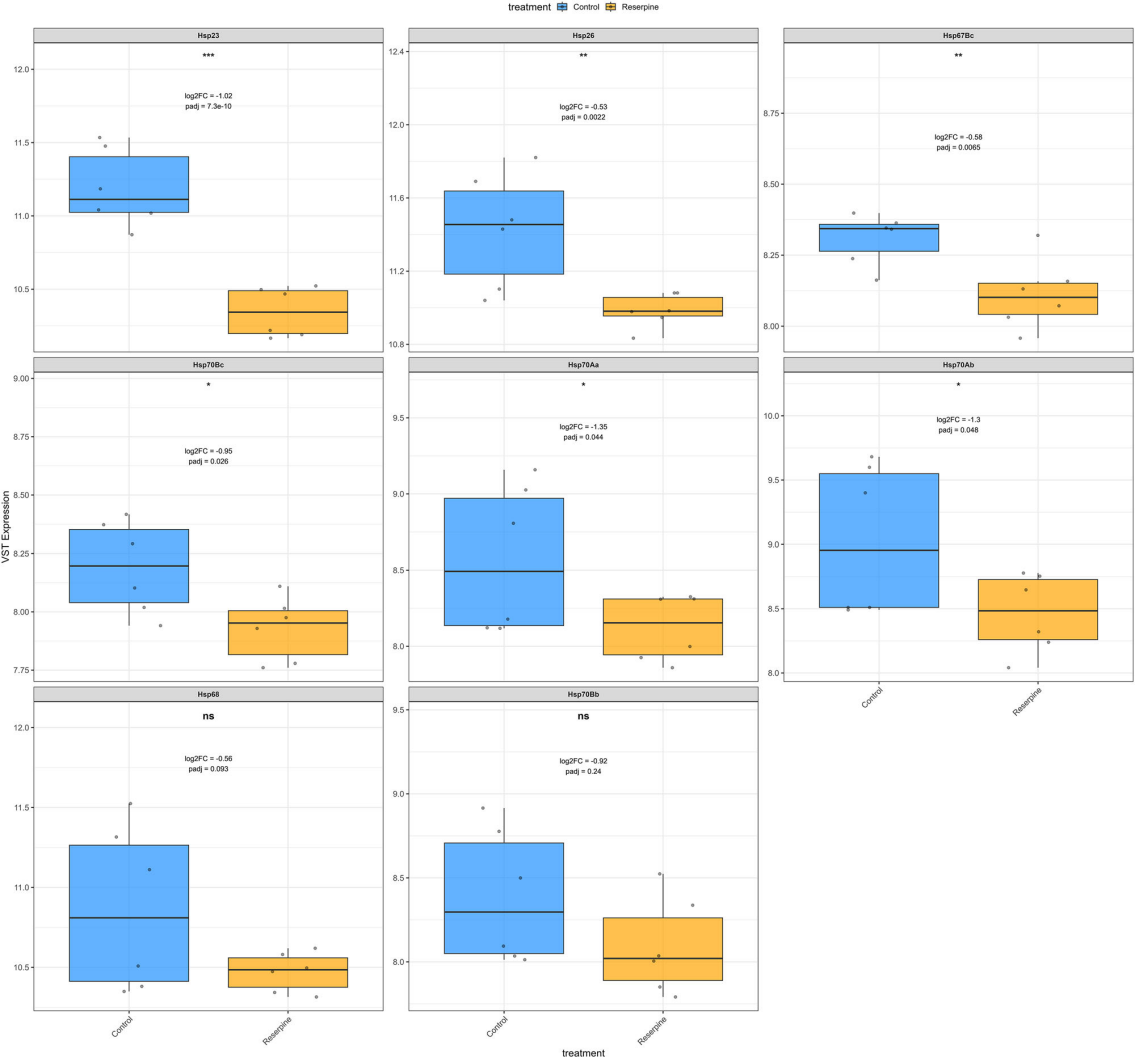
Expression of heat shock genes in young flies after heat shock. Box plots show variance-stabilized transformed (VST) mRNA expression levels of canonical *Drosophila* heat shock genes in young flies subjected to heat shock. Comparison is between control and reserpine-treated groups. Reserpine treatment leads to significant downregulation of heat shock gene expression. Annotations within each panel indicate the log2fold change, adjusted *p*-value, and difference in mean VST expression (ΔVST) between treatments. Only significant genes |log2FC| > 0.5 and padj < 0.05 are taken to be significant.

**Fig. 8:**
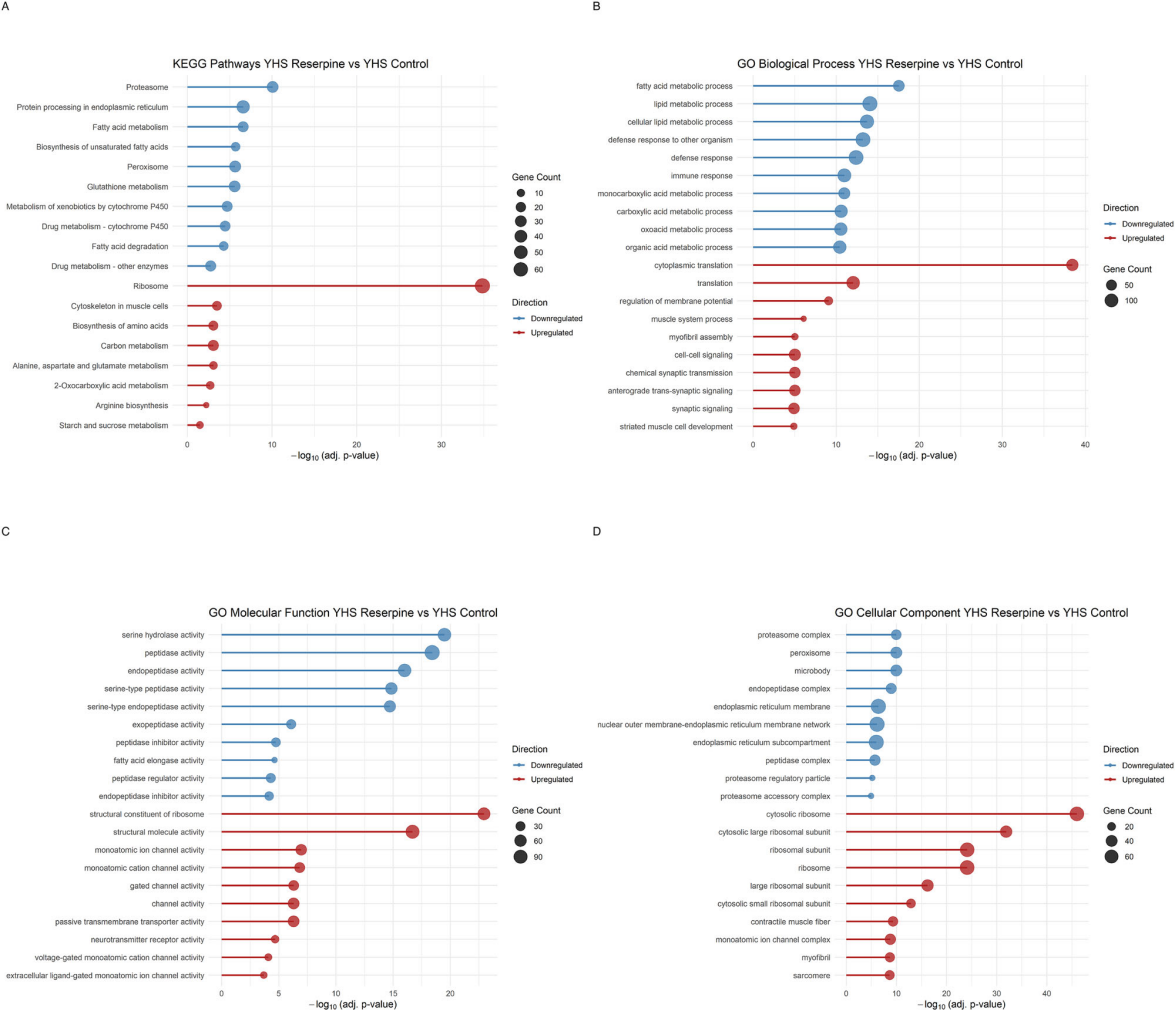
Over-representation analysis (ORA) of transcriptomic changes in reserpine-treated young flies under heat-stress (YHS Reserpine vs. YHS Control). **A** KEGG pathway enrichment; **B** GO Biological Process; **C** GO Molecular Function; **D** GO Cellular Component. Downregulated terms include fatty acid metabolism, proteasome, glutathione activity, and immune/metabolic processes, while upregulated terms include translation, biosynthesis, ion channel activity, and sarcomere-related components. Analyses were performed using clusterProfiler. Dot size indicates gene count, and position on the x-axis represents -log₁₀(adjusted *p*-value). Color indicates direction: blue downregulated, red upregulated.

In young flies subjected to heat shock, reserpine treatment resulted in significant suppression of several key heat shock response genes. Notably, *Hsp23*, *Hsp26*, *Hsp70Bc*, *Hsp70Aa*, and *Hsp70Ab* were all significantly downregulated in reserpine-treated flies compared to heat-shocked controls (Fig. 7). While *Hsp68* and *Hsp70Bb* were not significantly changed, they also showed a trend toward downregulation. These findings indicate that reserpine broadly impairs the transcriptional heat shock response, potentially compromising the fly’s capacity to manage proteotoxic stress. In flies, the maternal loading in embryos of Hsp23 mRNAs increases thermotolerance^58^ and loss of *Hsp70* Genes including *Hsp70Ab* reduces thermotolerance and survival^59^. Hsp70 group of genes (including *Hsp70Ab, Hsp70Bc, Hsp70Aa*) are among the most highly inducible and protective heat shock proteins in *Drosophila*, essential for survival under stress^59^. *Hsp83* (the Drosophila homolog of Hsp90) is involved in protein folding and stabilization of key signaling proteins^60^. Small Hsps (*Hsp23, Hsp26, Hsp27*) are rapidly induced by heat and oxidative stress and help protect cells from damage^61^. Overall, several key pathways include those involved in proteasome, fatty acid metabolism, glutathione, and cytochrome P450 detoxification (Figs. 8A, B, S4A, B). Peroxisome, lysosome, serine hydrolase and peptidase activity (Figs. 8C, D, S4C, D) were downregulated in Reserpine vs. control in young HS. Interestingly, these pathways, also suppressed in old treated flies, failed to upregulate in response to heat, compromising cellular defense and stress resilience as mentioned earlier. Simultaneously, there was a highly enriched upregulation of energetically costly processes such as ribosome and cytoplasmic translation, amino acid biosynthesis, synaptic signaling (Figs. 8A, B, S4A, B), muscle contraction, ion channel activity, respiratory chain complex in drug treated HS flies (Figs. 8C, D, S4C, D). This shift toward high-cost cellular activities may increase the vulnerability to heat-tress, as energy is diverted from protective mechanisms to processes that further strain cellular resources.

### Downregulation of known longevity genes in old flies

**Fig. 9:**
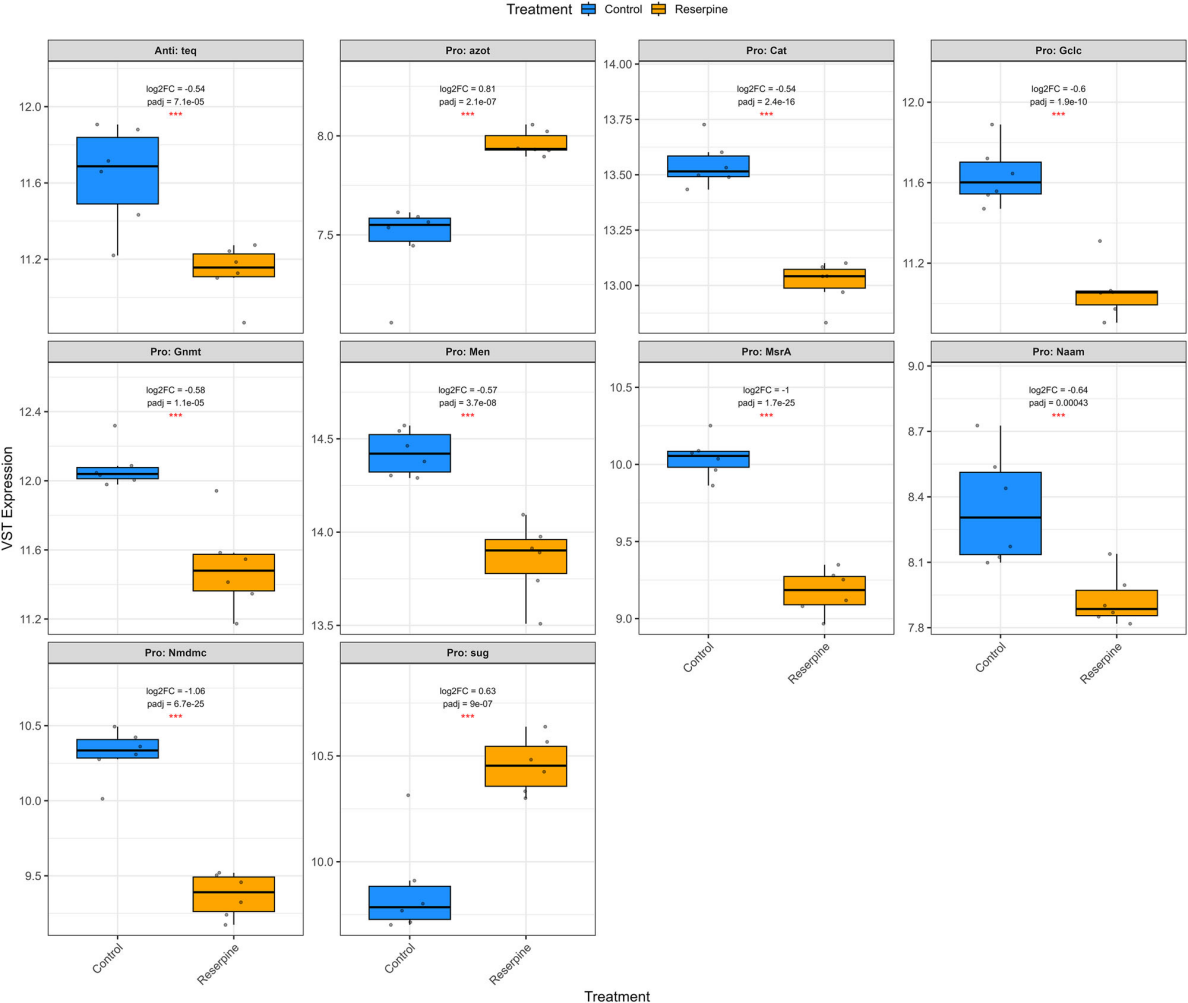
Differential expression of GenAge longevity-associated genes in old flies. Bar plots and clustered heatmaps depict significantly regulated GenAge genes (padj < 0.05, |VST difference| > 0.5). Many pro-longevity genes are downregulated in reserpine-treated flies, particularly in aged samples. Data represent normalized expression values.

Lastly, we compared our differentially expressed genes (DEGs) with the GenAge Model Organism (https://genomics.senescence.info/genes/index.html) dataset for *Drosophila melanogaster*, which compiles 2205 genes associated with aging or longevity based on genetic modification experiments reported in the literature. This curated set includes only genes that have a significant impact on lifespan and/or aging when genetically altered, while genes that reduce lifespan solely by inducing specific diseases without evidence of accelerated or premature aging are generally excluded to maintain a focus on the aging process. Within this dataset, genes are classified as either “anti-longevity” (*n* = 1101), which promote aging or reduce lifespan, or “pro-longevity” (*n* = 545), which delay aging or extend lifespan^62^. For *Drosophila melanogaster*, a total of 241 longevity genes with their function, longevity effect and study references were downloaded from their GenAge database website (https://genomics.senescence.info/genes/search.php?organism=Drosophila+melanogaster). We identified a consistent set of longevity-associated genes expression was significantly altered following reserpine treatment in aged Drosophila. To ensure both statistical and biological relevance, we filtered for genes that met the significance threshold (adjusted *p*-value < 0.05 and |logFC| > 0.5). Only GenAge-annotated genes passing both thresholds were retained for downstream visualization and interpretation (Fig. 9). A transcriptional suppression was observed in the old cohort, where reserpine treatment reduced expression of the nine pro-longevity genes, along with one anti-longevity gene. These annotations are based of anti and pro is based on 1–2 studies in the GenAge database so does not provide a full picture and many of these gene can change depending on context of the treatment (Fig. 9).

### qRT-PCR validation confirms metabolic reprogramming and suppression of stress defense genes

To validate the differential expression profiles identified by RNA-seq, we performed qRT-PCR on a subset of top-regulated targets in aged flies (old) and young flies subjected to heat-stress (Young HS). We selected candidate genes that were highly strong upregulation or downregulation in both young and old cohorts (Table S2) representing distinct functional categories implicated in the longevity trade-off, including carbohydrate metabolism (*Amyrel*)^63^, sulfotransferase activity (*St2*)^64^, and oxidative stress response gene which is involved in lifespan extension in fruit fly (*MsrA*)^65^ and nutrient storage*, Lsp1beta, Lsp2*)^66^.

In aged flies, Reserpine treatment (1000 µM) induced a transcriptional signature consistent with our RNA-seq data. We observed a robust upregulation of *Amyrel* and *St2* relative to age-matched controls (Fig. S5A). In contrast, genes associated with somatic maintenance were significantly downregulated. Most notably, *MsrA* (Methionine sulfoxide reductase A), a key enzyme for repairing oxidatively damaged proteins, was suppressed^63–65^.

To determine if this transcriptional signature correlates with the compromised stress resilience observed in Reserpine-treated animals, we assessed these targets in Young Heat-stress flies. The Reserpine-mediated signature was largely conserved: *Amyrel* and *St2* remained upregulated, while the protective factors *MsrA, Lsp1beta, Lsp2* were significantly downregulated (Fig. S5B). These trends validate the results from sequencing (Table S2).

### Common features in treated flies among both cohorts

**Fig. 10:**
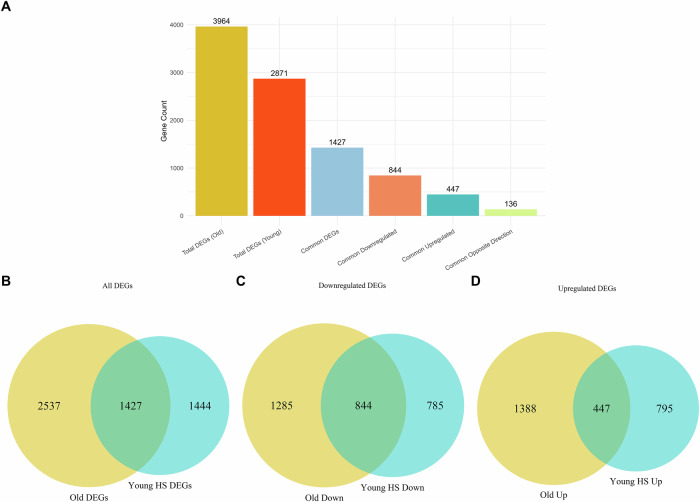
Shared transcriptional signatures between Reserpine-treated aged flies and young flies under heat-stress. **A** Bar plots showing common DEGs overall, up and down in both cases, and opposite in both cases. **B** Venn diagram showing overlap of all DEGs (padj < 0.05) between Reserpine Old vs. Control Old and Young Heat-Stress Reserpine vs. Young Heat-Stress Control. **C** Shared genes are separated into commonly downregulated subsets. **D** Shared genes are separated into commonly upregulated subsets.

There are several common features in old and young flies under reserpine treatment such as metabolic suppression in old and maladaptive upregulation due to heat-stress of processes that leads to lethality in heat shocked drug treated flies. Interestingly, we noticed that overlapping pathways were impacted by reserpine both in aged and in young flies heat-stress when treated with the drug. Venn diagram revealed that 1427 genes (padj <0.05) were differentially expressed in both the old and heat-stressed groups. Of those, 844 genes were commonly downregulated by reserpine treatment while 447 genes were upregulated in both groups. Notably, 136 DEGs were differentially regulated upon treatment (old and heat-stressed, but in opposite direction of regulation (Fig. 10A–D). Enrichment of the common upregulated and downregulated genes revealed common terms similar with overall enrichments (Fig. S6). Enrichment analyses thus confirm a broad suppression of protective, aging-linked pathways like observations in on old flies where terms like fatty acid, serine hydrolase and serine peptidase and glutathione metabolism, and metabolism via cytochrome P450 are downregulated in short treatment duration of 13 days and also in long-term treatment of 43 days.

## Discussion

**Fig. 11:**
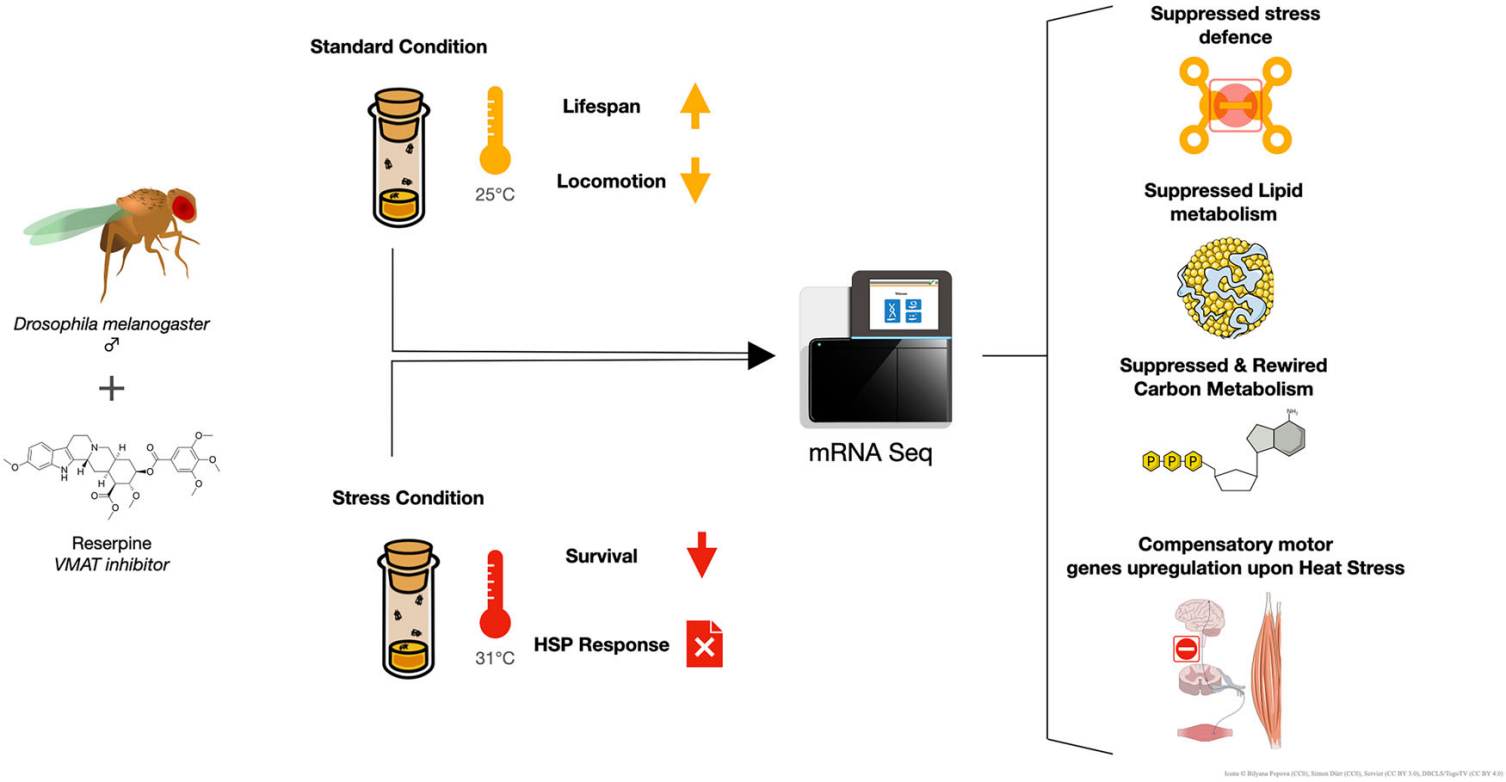
Schematic representation of the effects of Reserpine treatment and methods used. Flies fed with VMAT inhibitor Reserpine showed increased lifespan while impairing locomotion and heat-stress resilience. The transcriptome shifts to a rewired, energy preserving, immune-suppressed state.

Our study demonstrates that chronic dietary treatment with reserpine extends male *Drosophila* lifespan while simultaneously impairing locomotor activity, reducing survival under heat-stress, and suppressing metabolism and immune response including heat-shock response genes (Fig. 11). These results highlight a classic Geroscience trade-off: pharmacological interventions that enhance longevity may compromise stress resilience^28^ and physical vigor, particularly under challenging conditions.

Previous studies have shown that depletion of serotonin or octopamine can stimulate a “low food” signal, activating dietary restriction (DR) - like pathway responses and also modulating metabolism and stress tolerance via mechanisms such as neuron-gut signaling and p38-MAPK^67–69^. While mild depletion of either neurotransmitter may promote longevity by inducing metabolic restraint^67,69^ excessive loss impairs stress resilience including heat, hypoxia, and pathogen defense due to suppressed stress responses^69–71^. In our study, strongly downregulated metabolic and stress-response pathways including reduced heat-stress resistance in reserpine-treated flies are consistent with such monoamine neurotransmitter depletion-like states but via pharmacological VMAT inhibition, reflecting the trade-off between lifespan extension and stress vulnerability in treated flies. Reserpine’s classical action as a vesicular monoamine transporter (VMAT) inhibitor depletes stored catecholamines^20^ including octopamine which is epinephrine equivalent of insects (a key compound for “fight-or-flight” behaviors^72,73^. Therefore, reserpine’s impairment of heat-avoidance likely reflects a disrupted “fight-or-flight” response, providing a mechanistic basis for the impaired ability to avoid noxious heat^44^ and impaired thermotolerance and locomotor observed in our assays. In addition, depletion of dopamine in genetic DA-loss models shows impaired activity^74^ which mirrors reduced locomotion and motor neuron activity VMAT mutants^69,75^ reflecting in our findings with reduced climbing activity.

Our study identifies clear tradeoffs of reserpine-induced longevity, rendering the flies less active and displaying impaired heat-stress resilience. Reserpine-treated flies failed to mount a canonical heat shock response, with significant suppression of inducible heat shock proteins (HSPs) such as Hsp23, Hsp26, Hsp67Bc and others. HSPs are critical for thermotolerance, protein refolding, and cellular survival during proteotoxic stress and aging^50,58,59^. The suppression of detoxification pathways to activate essential adaptive responses contributes to increased mortality during heat exposure. As observed in an earlier study by Bressan et al.^44^, reserpine treatment significantly reduces heat-stress tolerance in *Drosophila melanogaster*, impairing their ability to avoid noxious heat and decreasing locomotor activity, likely through disruption of monoaminergic signaling pathways. This pharmacological intervention compromises the flies’ stress resilience, making them more susceptible to heat-induced damage^44^. Furthermore, in our study, immune, and inflammatory signaling components, including those related to innate immunity and lysosomal proteases, were suppressed. This aligns with the concept of inflammaging; whereby chronic low-grade immune activation contributes to tissue decline^76^.

Reserpine induced a pronounced transcriptomic shift in old as well as young HS cohorts, marked by broad suppression of metabolic, immune, and stress-response genes. Mechanistically, this suppressed metabolic state seems to be analogous to the suppression of mTOR, i.e., master regulator of cellular metabolism. Metabolic suppression via mTOR depletion is shown to promote longevity^77^, but in our study, it is achieved via neuromodulation through reserpine treatment. Consequently, with metabolic suppression, few pro-longevity genes were also downregulated in treated flies^62^ (Fig. 9). At first glance, this might seem counterintuitive, but it is known that pro-longevity genes can be related to stress-resistance or antioxidant genes^23^. Antioxidant gene suppression might reflect a reduced demand for oxidative stress defense due to broad reduction in metabolic load, similar to mTOR suppression or sustained caloric restriction^77–79^. This can be observed as downregulated carbonic and fatty acids metabolism enrichment (Fig. 5A, B). Reduced energy demand and/or need is also reflected in reduced activity of flies in climbing assays (Fig. 2).

Interestingly, two key pathways that were enriched in downregulated terms by reserpine treatment in both old and young HS group were serine metabolism (hydrolase and peptidase) and glutathione transferase (GST) activity. Serine supplementation in *C. elegans* and yeast has been shown to increase lifespan, while in pre-clinical rodent studies show reductions in both circulating and hippocampal serine levels with age^57^. Aging in *Drosophila* is linked to shift from glycolysis to serine metabolism and purine metabolism. In contrast, long-lived models such as PRC2 mutants maintain youthful serine metabolism and avoid such metabolic shifts^49,54^, suggesting that reducing serine peptidase or hydrolase activity may be longevity-promoting by allowing higher serine availability by suppressing its metabolism. Similarly, suppressed glutathione transferase (GST) activity which is central to oxidative stress defense^80^, indicates immune suppression which could indicate less oxidative burden or alternative antioxidant mechanisms in play. Reserpine’s ability to suppress this immune-metabolic axis may be central to its pro-longevity effect. Collectively, these point to a suppression of energy-intensive and stress-inducing catabolic processes, often upregulated during aging and chronic immune activation^1,81^.

In contrast to the widespread metabolic suppression, key glycolytic and mitochondrial metabolic pathways were mildly upregulated, including Glycolysis/Gluconeogenesis, TCA cycle, and Amino acid biosynthesis. These shifts may reflect a reorganization of mitochondrial energy metabolism toward substrates such as carbohydrates and selected amino acids, with reduced reliance on fatty acid β-oxidation or branched-chain amino acid catabolism both of which generate greater oxidative stress^82^. Many common suppressions in both young HS and old group are shown in Fig. S6, like Peroxisome, Glutathione metabolism, Drug and Xenobiotic metabolism by Cytochrome P450, Antioxidant activity. In old flies, a major but weakly upregulated category enriched across GO CC and GO BP is cilium, and cilium-dependent cell motility, insemination, and courtship behavior in treatment group. These enrichments hint at reserpine preserving or reactivating neurological or sensory-motor capacity in aged flies. Given that cilia are central to chemosensation and behavioral circuits in *Drosophila*^83,84^, this may reflect a compensatory mechanism for maintenance of behavior vigor in context of chemosensation and response to stimuli, not simply reproductive investment due to reduced activity in treated flies.

From the list of gene compared (Table S1), we found that in both old and young heat-shocked (YHS) flies, *Act88F*, a gene encoding an indirect flight muscle actin^85^, was significantly upregulated in reserpine-treated flies indicating a consistent transcriptional response related to muscle remodeling^86^. Another gene, *TpnC41C* (Troponin C at 41C), encodes a muscle-specific protein primarily expressed in the jump muscles^87^ is upregulated in both young HS and old flies. Both genes’ upregulation could be a compensatory mechanism to reduced locomotor ability. Moreover, *mt: ND3* (mitochondrial NADH-ubiquinone oxidoreductase chain 3) which is subunit of complex I of the mitochondrial respiratory chain involved in energy metabolism^88^, was also significantly downregulated, potentially reflecting age-related susceptibility of mitochondrial function to monoaminergic disruption in old flies. Suppression of complex 1 of the Electron transport chain is associated with longevity in oocytes by avoiding ROS generation^89^. Genes like *ple* which encodes a tyrosine hydroxylase^90^, the first and rate-limiting step in the synthesis of dopamine^91^ and *TyrR* when overexpressed has neuromodulatory effect of male courtship behavior leading to high mating behavior^92^.

Taken together, the transcriptomic profiles demonstrate that reserpine triggers a marked, condition-specific suppression of metabolic, immune, and stress-response pathways in both aged and heat-stressed flies. This effect is reflected in the consistent downregulation of key genes and the clear separation of transcriptomic signatures between treated and control groups, thus supporting the conclusion that reserpine promotes a immune suppressed and low-energy expenditure state in flies under both physiological aging and acute stress conditions. While this study focused on flies, similar observations with reserpine have been made in worms. Notably, Saharia et al.^3^ showed that reserpine can extend worm lifespan through pathways that modulate acetylcholine release. In worms, the effect was independent of the VMAT ortholog (cat-1) and instead required dop-3 (a D2-type dopamine receptor) and eri-1 (an exoribonuclease), which act in parallel to reduce acetylcholine release and thereby increase lifespan. These findings highlight that reserpine-mediated longevity in worms is not obligatorily linked to VMAT inhibition but arises from altered presynaptic neurotransmission^3^.

However, our findings indicate that in flies, reserpine acts primarily through suppression of metabolic and inflammatory stress, coupled with energy reprogramming. This may highlight species-specific divergence in the molecular architecture of lifespan regulation. In *Drosophila*, our study, the first to examine reserpine’s aging effects in flies demonstrates that dVMAT inhibition and consequent monoamine depletion underlie both longevity extension and altered stress responses, consistent with prior reports linking VMAT suppression to altered behavioral states^37,38^.

These observations underscore an important conceptual point: reserpine perturbs monoaminergic signaling across species, a conserved pharmacological entry point but the downstream “wiring” to lifespan regulation may be species-specific. This is exemplified by antagonistic stress tolerance outcomes, where reserpine increases thermotolerance in worms^13^ but reduces thermotolerance in flies^44^ (Fig. 3). Such differences suggest that while the VMAT inhibition is conserved, the downstream physiological outputs diverge.

Our data therefore support VMAT inhibition as the proximate mechanism underlying the locomotor and thermotolerance phenotypes. However, whether the observed lifespan extension depends directly on VMAT inhibition remains unresolved. It is possible that longevity arises from VMAT inhibition and monoamine depletion, but an alternative explanation could be that, as in worms, partially VMAT-independent mechanisms contribute to lifespan benefits^3^, while VMAT inhibition primarily drives the observed locomotor and stress phenotypes in flies.

Despite mechanistic differences, several evolutionarily conserved features support therapeutic translation: (1) VMAT function is conserved across species; *C. elegans* cat-1 (49% identical to mammalian VMAT2) to fly dVMAT to human VMAT2^93^, preserving pharmacological sensitivity to reserpine observed by multiple studies; (2) Dopamine receptor signaling influences lifespan across invertebrates^94,95^ and at least signaling is influenced by aging in vertebrates^96,97^, with human DRD2 polymorphisms associated with memory performance with aging^98^; (3) stress-response pathways including heat shock protein induction are modulated by reserpine in both models worms and flies (antagonistically)^13^; and (4) metabolic remodeling of monoamine metabolism occurs across worms, flies, and mammals with aging^6^ and aging and lifespan is regulated by neural excitation^4^.

Our study has several limitations. Firstly, our focus was on male flies only therefore sex‑specific effects on thermotolerance, and reserpine response remain to be tested. We also did not determine reproductive output in females, which are often affected by monoamine depletion and may contribute to the observed trade-offs. Finally, while our RNA-seq provides a snapshot of global gene expression, complementary proteomic and metabolomic data would provide a more complete picture of the molecular consequences of reserpine treatment. While our data strongly implicate VMAT inhibition and monoamine depletion in reserpine’s effects on locomotion, stress sensitivity, and lifespan, we cannot exclude the possibility that lifespan extension also involves VMAT-independent mechanisms, as reported in *C. elegans*. Disentangling whether longevity and locomotor/stress phenotypes share a common VMAT-dependent origin, or represent separable pathways, will require targeted genetic analyses such as dVMAT mutants and tissue-specific rescue.

Mechanistically, future studies should dissect which monoaminergic circuits dopaminergic, serotonergic, or octopaminergic are most critical for mediating reserpine’s effects specific to Fruit flies. Targeted manipulation of these pathways may enable the development of interventions that maximize longevity benefits while minimizing adverse effects. To achieve this, comprehensive phenotyping including behavioral, metabolic, and reproductive assays should be integrated with transcriptomic and proteomic analyses to elucidate the systemic consequences of chronic monoamine depletion. Cross-species validation, particularly in mammalian models, will be essential to assess the translational potential and safety of reserpine-related compounds. For human applications, this multi-pathway architecture suggests that clinically available monoaminergic signaling modulators could engage the evolutionarily conserved monoaminergic axis. Since reserpine acts on VMAT; it results in elimination of multiple neurotransmitter stores (dopamine, serotonin, norepinephrine and histamine)^20^ affecting multiple downstream pathways, therefore a more specific target drug could be studied further as some candidate hypertension drugs have already shown lifespan extension in worms and influence aging in humans^11–14^. Given the context-dependent nature of reserpine’s effects, systematic investigation of dose-response relationships, intervention timing will be crucial. In addition, as a common challenge in many studies, we cannot rule out that reserpine-induced changes in food palatability or feeding behavior may have resulted in mild dietary restriction, which itself can influence lifespan and stress resistance^99^. These parameters influence the balance between lifespan extension and health span, as observed with several other agents influencing metabolism such as anti-diabetic and anti-obesity drugs like metformin or potentially with semaglutide-based drugs where lifespan extension almost always comes with tradeoffs^100,101^.

In summary, reserpine’s effects in *Drosophila* exhibit robust lifespan extension coupled with locomotor and stress phenotypes consistent with VMAT inhibition. By isolating beneficial mechanisms and refining pharmacological approaches, it may be possible to promote healthy lifespan extension while minimizing adverse outcomes.

## Methods

### Fly husbandry and diet preparation

*Drosophila melanogaster* (Canton-S wild type) were reared at 25 °C, 60% humidity, under a 12:12 h light:dark cycle on standard corn meal with supplemented dry yeast vials with crosses containing (8:8::♂:♀). Overnight egg lay were performed for the crosses and parents were discarded next day. Standard fly food was prepared with a standardized cornmeal–soy–molasses medium (containing 3.21 L distilled water, 30 g soy flour, 260 g corn flour, 60 g active dry yeast, 26 g agar, 260 g molasses, and 130 g malt extract per batch), homogenized and autoclaved at 121 °C for 20 min. After cooling to ~60 °C, 83 mL of 10% (w/v) methylparaben (in ethanol) and 59 mL acid mix (60 mL 85% phosphoric acid, 418 mL propionic acid, distilled water to 2 L) were added, and the medium was dispensed aseptically (8 mL per vial) and allowed to solidify.

For all experiments, adult male flies (1 day post‑eclosion) were used to minimize variability from female reproductive status and to standardize aging comparisons. Flies were sexed under anesthesia using brief CO_2_ exposure and placed carefully in horizontally oriented vials to recover for around 15–20 min before putting them vertically inside incubator.

For reserpine containing food, Reserpine (Sigma-Aldrich, Cat. No. 83580; MW 608.68 g/mol) was dissolved in DMSO (Dimethyl sulphoxide, Carl ROTH; 250 N.1) to generate a 100 mM stock solution (760 mg in 12.5 mL DMSO). After autoclaving and cooling the standard fly food to approximately 60 °C, reserpine was added to achieve final concentrations of 0 (DMSO control), 200, 800, 1000, or 1500 μM, as indicated for each experiment.

### Survival assays

For survival assays under standard conditions, 1 day post-eclosion flies grown in standard corn meal media were pooled from and were housed in three biological replicates per drug concentration with *n* ~ 35 flies per vial and *N* ~ 105 flies per drug condition. Flies were maintained at 25 °C and 60% humidity fly incubator on reserpine-supplemented media and flipped on every alternate day along with recording dead flies. For pilot standard survival experiments, the fly were flipped and recorded three times a week.

### Heat-stress survival assay

For heat-stress survival experiments, flies were reared in standard condition as described above. One-day-old male flies were sexed under anesthesia using CO_2_ pads and housed in three biological replicates of 35 flies in each vial containing reserpine-supplemented food for 12 days at 25 °C and 60% humidity. On the 13th day, the flies were placed inside incubator (10-liter incubator (VWR® INCU-Line® IL 10) set at 31 °C untill all flies perished. Due to budget and space constraints, this incubator (separate from the main 25 °C, 60% humidity incubator) has no inbuilt humidity control. To maintain moisture during heat exposure, one vial filled distilled water was placed inside the incubator along with fly vials, helping to preserve ambient humidity along with moisture from the food medium. Food vials were replaced daily to ensure continuous nutrition and hydration, and flies were flipped daily to fresh vials to avoid waste accumulation and starvation or dehydration. Control and treated groups were handled identically, including brief removal at room temperature for survival scoring and vial changes. These measures aimed to provide a physiologically relevant heat-stress environment while minimizing confounding factors such as desiccation or starvation.

### Climbing assay

To evaluate locomotor function, a negative geotaxis climbing assay was performed on adult *Drosophila melanogaster* following chronic drug exposure. Flies were maintained on food containing 0 µM (control), 1000 µM, or 1500 µM of the test compound for 12 consecutive days, and the climbing assay was conducted on day 13th. In pilot survival experiments, reserpine treatment’s effect on locomotion were apparent by days 4–5; however, day 13th was chosen as a robust endpoint that captures early adulthood after which slow climbing ability decline starts at day 14 at 25 °C^102^.

Each treatment group consisted of six biological replicates, with *n* ~ 25–30 flies per replicate. For the assay, flies were gently transferred into clean, transparent plastic vials measuring 9 cm in height, pre-marked into five equal quadrants (each 1.8 cm high) based on previous study by Peleg et al.^42^ including the scoring system Weighted Climbing Index (WCI)^42^. To initiate the assay, flies containing vials were tapped to the table top at an exact marked spot three times in rapid succession to standardize the starting position and waited for them to climb while recording using a fixed phone camera on stand. Each trial was video recorded for 30 s, and the climbing position of flies was scored at the 10-s mark after 3 taps. The number of flies in each of the five vertical quadrants was recorded using video playback later. The experiments were performed in fly lab ambient lighting with a light source over herd and 25 ± 1 °C.

A Weighted Climbing Index (WCI) was calculated for each replicate using Eq. (1):

1 $${\bf{WCI}}=\frac{\left({\bf{q}}{\bf{1}}\times {\bf{1}}\right)+\left({\bf{q}}{\bf{2}}\times {\bf{2}}\right)+\left({\bf{q}}{\bf{3}}\times {\bf{3}}\right)+\left({\bf{q}}{\bf{4}}\times {\bf{4}}\right)+\left({\bf{q}}{\bf{5}}\times {\bf{5}}\right)}{{\bf{Total\; number\; of\; flies}}}$$

where qi is the number of flies present in the *i*th quadrant from bottom (1) to top (5) of the climbing vial. A higher WCI indicates better climbing performance, as more flies reach higher quadrants.

Statistical analysis was performed in GraphPad Prism using one-way ANOVA followed by Tukey’s multiple comparisons test, comparing all treatment groups. Results are presented as mean ± SEM, with significance set at *p* < 0.05.

### RNA extraction and sequencing

Flies were reared in standard condition as described above. One-day-old male flies were sexed under anesthesia using CO_2_ pads: Flies were collected at the following time points: old (43 days), and after young heat-stress (12 days at 25 °C followed by 24 h at 31 °C). Collected 1-day-old male flies were always in fly food supplemented with 1000 µM Reserpine till flash freezing in liquid nitrogen in both cohorts of old and young heat-stress. 10 flies per biological replicate *n* = 6 in 1.5 mL microcentrifuge tube were used.

Total RNA was extracted using the Monarch Total RNA Miniprep Kit (NEB T2010S). The RNA concentration was analyzed using NanoDrop and the RNA integrity was assessed on the 2100 Bioanalyzer system using an Agilent RNA 6000 Nano Kit (Agilent Technologies). Strand-specific mRNA libraries were prepared from 1 μg total RNA using the Illumina Stranded mRNA Prep Ligation kit according to the manufacturer’s recommendations (Illumina). Library quality and fragment size were verified using Agilent DNA 1000 Kit (Agilent Technologies). Libraries were quantified using a Qubit dsDNA HS Assay Kit (Invitrogen), normalized to 10 nM, pooled, denatured to 750 pM, and paired-end sequenced (2 × 101 bp) on the NextSeq 2000 system using P3 flowcell (Illumina) at the FBN sequencing facility, Dummerstorf, Germany.

### Quantitative real-time PCR (qRT-PCR)

RNA was extracted and analyzed as described before and same RNA material from *n* = 5 biological replicates (out of 6 used for sequencing) were used for qRT-PCR assay. 10 ng of total RNA was used for cDNA synthesis using the SensiFAST cDNA Synthesis Kit (Biocat, Cat. No. BIO-65053). Quantitative PCR was performed on a LightCycler 96 System (Roche) using the 2x SensiFast SYBR No-ROX Mix (Biocat, Cat. No. BIO-98005). Due to differences in the expression of housekeeping gene due to treatment (reserpine and/or heat-stress), *Rpl32* was used for Old cohort and *Gapdh* was used for Young HS for as reference housekeeping genes.

The thermal cycling conditions were: preincubation at 95 °C for 60 s, followed by 40 cycles of amplification (95 °C for 15 s, 60 °C for 15 s, 72 °C for 20 s), and a final melting curve analysis (95 °C to 97 °C) to confirm amplicon specificity. Relative gene expression was calculated using the 2−ΔΔCt method.

Primer sequences and annealing temperature are listed supplementary Table s3.

### Data analysis

Raw sequencing data (BCL files) were converted to FASTQ format using DRAGEN BCL Convert v3.10.11. Quality assessment was performed with FastQC v0.11.9. Adapter trimming, low-quality read filtering (mean Phred <20), and removal of reads <20 bp were performed using Trim Galore v0.6.10. Reads were aligned to the D. melanogaster BDGP6.46 reference genome (Ensembl r113) using HISAT2 v2.2.1. Gene-level counts were generated using HTSeq v2.0.2 (“union” mode), and genes with fewer than 10 total counts were excluded. Count matrices were imported into R via tximport and analyzed with DESeq2 v1.32.0. Data were normalized using the median-of-ratios method, and variance-stabilizing transformation was applied. Differential expression was tested using Wald tests with Benjamini-Hochberg multiple testing correction (padj <0.05). Differentially expressed genes (DEGs; padj < 0.05) were annotated and subjected to functional enrichment analyses using clusterProfiler v4.0, DOSE, enrichplot, and org.Dm.eg.db. Over-Representation Analysis (ORA) was performed for GO (Biological Process, Cellular Component, Molecular Function) and KEGG pathway terms using enrichGO() and enrichKEGG() functions (BH-adjusted *p* < 0.05; minGSSize = 10; maxGSSize = 500). Gene Set Enrichment Analysis (GSEA) was conducted using ranked log2 fold-changes.

Data visualization was carried out using ggplot2, including volcano plots and dot plots. Box plots were used to visualize expression differences for selected gene sets (GenAge, Heat Shock Genes) using VST expression and annotated with padj and |logFC| values. Genes were considered biologically relevant if they met both statistical (padj < 0.05) and expression (|logFC| ≥ 0.5) thresholds.

### Statistical analysis

All statistical analyses were conducted using R version 4.4.3 and GraphPad Prism (10.6.1). Survival data were analyzed using Kaplan–Meier survival curves, with log-rank tests used for group comparisons in R. Cox proportional hazards models, stratified by replicate, were used to calculate hazard ratios (HR) and 95% confidence intervals (CI).

For RNA-seq data, differential gene expression was assessed using the Wald test, and statistical significance was determined using the Benjamini-Hochberg correction for multiple testing, with a false discovery rate threshold of padj < 0.05.

Climbing assay performance was evaluated using the Weighted Climbing Index (WCI). WCI values were analyzed in GraphPad Prism using one-way ANOVA, followed by Tukey’s multiple comparisons test to determine statistically significant differences between treatment groups.

Statistical analyses for qRT-PCR were performed using GraphPad Prism (Version 10.6.1). To determine differential expression of the six target genes between Control (0 µM) and Reserpine (1000 µM) groups for Old and young HS, we performed multiple unpaired t-tests (one per gene) without assuming equal variances. Statistical significance was determined using multiple unpaired t-tests with False Discovery Rate (FDR) correction (Two-stage Benjamini, Krieger, and Yekutieli method; q < 0.05).

## Supplementary information

Supplementary Information

## Data Availability

Raw fastq files and metadata are available in the ArrayExpress database (http://www.ebi.ac.uk/arrayexpress) under accession number E-MTAB-15429 (https://www.ebi.ac.uk/biostudies/ArrayExpress/studies/E-MTAB-15429?key=255702e5-c660-407a-9cd9-cdb2ea27ed35).

## References

[1] Lopez-Otin, C., Blasco, M. A., Partridge, L., Serrano, M. & Kroemer, G. Hallmarks of aging: an expanding universe. *Cell* **186**, 243–278 (2023). 36599349 10.1016/j.cell.2022.11.001

[2] Costa-Machado, L. F. et al. Peripheral modulation of antidepressant targets MAO-B and GABAAR by harmol induces mitohormesis and delays aging in preclinical models. *Nat. Commun.* **14**, 2779 (2023). 37188705 10.1038/s41467-023-38410-y PMC10185515

[3] Saharia, K., Kumar, R., Gupta, K., Mishra, S. & Subramaniam, J. R. Reserpine requires the D2-type receptor, dop-3, and the exoribonuclease, eri-1, to extend the lifespan in C. elegans. *J. Biosci.* **41**, 689–695 (2016). 27966489 10.1007/s12038-016-9652-7

[4] Zullo, J. M. et al. Regulation of lifespan by neural excitation and REST. *Nature* **574**, 359–364 (2019). 31619788 10.1038/s41586-019-1647-8 PMC6893853

[5] Kenyon, C. J. The genetics of ageing. *Nature* **464**, 504–512 (2010). 20336132 10.1038/nature08980

[6] Santin, Y., Resta, J., Parini, A. & Mialet-Perez, J. Monoamine oxidases in age-associated diseases: new perspectives for old enzymes. *Ageing Res. Rev.* **66**, 101256 (2021). 33434685 10.1016/j.arr.2021.101256

[7] Madeo, F., Carmona-Gutierrez, D., Hofer, S. J. & Kroemer, G. Caloric restriction mimetics against age-associated disease: targets, mechanisms, and therapeutic potential. *Cell Metab.* **29**, 592–610 (2019). 30840912 10.1016/j.cmet.2019.01.018

[8] Hofer, S. J., Davinelli, S., Bergmann, M., Scapagnini, G. & Madeo, F. Caloric restriction mimetics in nutrition and clinical trials. *Front Nutr.* **8**, 717343 (2021). 34552954 10.3389/fnut.2021.717343 PMC8450594

[9] Snell, T. W. et al. Repurposing FDA-approved drugs for anti-aging therapies. *Biogerontology* **17**, 907–920 (2016). 27484416 10.1007/s10522-016-9660-x PMC5065615

[10] Kulkarni, A. S. et al. Geroscience-guided repurposing of FDA-approved drugs to target aging: a proposed process and prioritization. *Aging Cell* **21**, e13596 (2022). 35343051 10.1111/acel.13596 PMC9009114

[11] Bennett, D. F. et al. Rilmenidine extends lifespan and healthspan in *Caenorhabditis elegans* via a nischarin I1-imidazoline receptor. *Aging Cell* **22**, e13774 (2023). 36670049 10.1111/acel.13774 PMC9924948

[12] Ito, A. et al. Metolazone upregulates mitochondrial chaperones and extends lifespan in *Caenorhabditis elegans*. *Biogerontology* **22**, 119–131 (2021). 33216250 10.1007/s10522-020-09907-6

[13] Srivastava, D. et al. Reserpine can confer stress tolerance and lifespan extension in the nematode C. elegans. *Biogerontology* **9**, 309–316 (2008). 18409080 10.1007/s10522-008-9139-5

[14] Tang, B. et al. Longitudinal associations between use of antihypertensive, antidiabetic, and lipid-lowering medications and biological aging. *Geroscience* **45**, 2065–2078 (2023). 37032369 10.1007/s11357-023-00784-8 PMC10400489

[15] Gao, X. et al. Accelerated DNA methylation age and the use of antihypertensive medication among older adults. *Aging* **10**, 3210–3228 (2018). 30414594 10.18632/aging.101626 PMC6286862

[16] Shamon, S. D. & Perez, M. I. Blood pressure-lowering efficacy of reserpine for primary hypertension. *Cochrane Database Syst. Rev.* **12**, CD007655 (2016). 27997978 10.1002/14651858.CD007655.pub3 PMC6464022

[17] Wang, Y. et al. Transport and inhibition mechanism for VMAT2-mediated synaptic vesicle loading of monoamines. *Cell Res.* **34**, 47–57 (2024). 38163846 10.1038/s41422-023-00906-z PMC10770148

[18] Raguraman, S. et al. Reserpine induces apoptosis in drug-resistant cancer through modulating STAT3 and NF-κB signaling. *Indian J. Pharm. Educ. Res.* **58**, s900–s909 (2024).

[19] Kapur, S., Zipursky, R., Jones, C., Remington, G. & Houle, S. Relationship between dopamine D(2) occupancy, clinical response, and side effects: a double-blind PET study of first-episode schizophrenia. *Am. J. Psychiatry* **157**, 514–520 (2000). 10739409 10.1176/appi.ajp.157.4.514

[20] Strawbridge, R., Javed, R. R., Cave, J., Jauhar, S. & Young, A. H. The effects of reserpine on depression: a systematic review. *J. Psychopharmacol.* **37**, 248–260 (2023). 36000248 10.1177/02698811221115762 PMC10076328

[21] Siddiqui, M., Bhatt, H., Judd, E. K., Oparil, S. & Calhoun, D. A. Reserpine substantially lowers blood pressure in patients with refractory hypertension: a proof-of-concept study. *Am. J. Hypertens.* **33**, 741–747 (2020). 32179903 10.1093/ajh/hpaa042 PMC7402229

[22] Weir, M. R. Reserpine: a new consideration of and old drug for refractory hypertension. *Am. J. Hypertens.* **33**, 708–710 (2020). 32303749 10.1093/ajh/hpaa069 PMC7402223

[23] Soo, S. K. et al. Biological resilience and aging: activation of stress response pathways contributes to lifespan extension. *Ageing Res. Rev.* **88**, 101941 (2023). 37127095 10.1016/j.arr.2023.101941

[24] Soo, S. K. et al. Genetic basis of enhanced stress resistance in long-lived mutants highlights key role of innate immunity in determining longevity. *Aging Cell* **22**, e13740 (2023). 36514863 10.1111/acel.13740 PMC9924947

[25] Kharat, P. et al. Ellagic acid prolongs the lifespan of *Drosophila melanogaster*. *Geroscience* **42**, 271–285 (2020). 31786733 10.1007/s11357-019-00135-6 PMC7031466

[26] Johnson, T. E. et al. Relationship between increased longevity and stress resistance as assessed through gerontogene mutations in Caenorhabditis elegans. *Exp. Gerontol.* **36**, 1609–1617 (2001). 11672983 10.1016/s0531-5565(01)00144-9

[27] Ukraintseva, S. et al. Decline in biological resilience as key manifestation of aging: potential mechanisms and role in health and longevity. *Mech. Ageing Dev.* **194**, 111418 (2021). 33340523 10.1016/j.mad.2020.111418 PMC7882032

[28] Huffman, D. M., Schafer, M. J. & LeBrasseur, N. K. Energetic interventions for healthspan and resiliency with aging. *Exp. Gerontol.* **86**, 73–83 (2016). 27260561 10.1016/j.exger.2016.05.012 PMC5133182

[29] Venkatasubramani, A. V. et al. The fruit fly acetyltransferase chameau promotes starvation resilience at the expense of longevity. *EMBO Rep.* **24**, e57023 (2023). 37724628 10.15252/embr.202357023 PMC10561354

[30] Martin, C. A. & Krantz, D. E. *Drosophila melanogaster* as a genetic model system to study neurotransmitter transporters. *Neurochem. Int.* **73**, 71–88 (2014). 24704795 10.1016/j.neuint.2014.03.015 PMC4264877

[31] Deshpande, S. A., Freyberg, Z., Lawal, H. O. & Krantz, D. E. Vesicular neurotransmitter transporters in *Drosophila melanogaster*. *Biochim. Biophys. Acta Biomembr.* **1862**, 183308 (2020). 32305263 10.1016/j.bbamem.2020.183308 PMC7508792

[32] McKinney, H. M., Sherer, L. M., Williams, J. L., Certel, S. J. & Stowers, R. S. Characterization of Drosophila octopamine receptor neuronal expression using MiMIC-converted Gal4 lines. *J. Comp. Neurol.* **528**, 2174–2194 (2020). 32060912 10.1002/cne.24883 PMC7998515

[33] Kume, K., Kume, S., Park, S. K., Hirsh, J. & Jackson, F. R. Dopamine is a regulator of arousal in the fruit fly. *J. Neurosci.* **25**, 7377–7384 (2005). 16093388 10.1523/JNEUROSCI.2048-05.2005 PMC6725300

[34] Eriksson, A. et al. Neuromodulatory circuit effects on Drosophila feeding behaviour and metabolism. *Sci. Rep.* **7**, 8839 (2017). 28821829 10.1038/s41598-017-08466-0 PMC5562903

[35] Knapp, E. M. et al. Mutation of the *Drosophila melanogaster* serotonin transporter dSERT impacts sleep, courtship, and feeding behaviors. *PLoS Genet* **18**, e1010289 (2022). 36409783 10.1371/journal.pgen.1010289 PMC9721485

[36] Greer, C. L. et al. A splice variant of the Drosophila vesicular monoamine transporter contains a conserved trafficking domain and functions in the storage of dopamine, serotonin, and octopamine. *J. Neurobiol.* **64**, 239–258 (2005). 15849736 10.1002/neu.20146

[37] Nall, A. H. & Sehgal, A. Small-molecule screen in adult *Drosophil*a identifies VMAT as a regulator of sleep. *J. Neurosci.* **33**, 8534–8540 (2013). 23658190 10.1523/JNEUROSCI.0253-13.2013 PMC3677510

[38] Chang, H. Y. et al. Overexpression of the Drosophila vesicular monoamine transporter increases motor activity and courtship but decreases the behavioral response to cocaine. *Mol. Psychiatry* **11**, 99–113 (2006). 16189511 10.1038/sj.mp.4001742

[39] Piper, M. D. W. & Partridge, L. Drosophila as a model for ageing. *Biochim. Biophys. Acta Mol. Basis Dis.* **1864**, 2707–2717 (2018). 28964875 10.1016/j.bbadis.2017.09.016

[40] Lee, S.-H. & Min, K.-J. *Drosophila melanogaster* as a model system in the study of pharmacological interventions in aging. *Transl. Med. Aging* **3**, 98–103 (2019).

[41] Tsurumi, A. & Li, W. X. Aging mechanisms—a perspective mostly from Drosophila. *Adv. Genet* **1**, e10026 (2020). 36619249 10.1002/ggn2.10026 PMC9744567

[42] Peleg, S. et al. Life span extension by targeting a link between metabolism and histone acetylation in Drosophila. *EMBO Rep.* **17**, 455–469 (2016). 26781291 10.15252/embr.201541132 PMC4772992

[43] Alruiz, J. M., Peralta-Maraver, I., Bozinovic, F., Santos, M. & Rezende, E. L. Thermal tolerance in Drosophila: Repercussions for distribution, community coexistence and responses to climate change. *J. Anim. Ecol.* **91**, 655–667 (2022). 34951017 10.1111/1365-2656.13653

[44] Bressan, G. N., Cardoso, P. M., Reckziegel, J. & Fachinetto, R. Reserpine and PCPA reduce heat tolerance in *Drosophila melanogaster*. *Life Sci.* **318**, 121497 (2023). 36780938 10.1016/j.lfs.2023.121497

[45] Jiang, Y. et al. Monoamine neurotransmitters control basic emotions and affect major depressive disorders. *Pharmaceuticals* **15**, 1203 (2022). 36297314 10.3390/ph15101203 PMC9611768

[46] Flügge, G., Van Kampen, M. & Mijnster, M. J. Perturbations in brain monoamine systems during stress. *Cell Tissue Res.* **315**, 1–14 (2004). 14579145 10.1007/s00441-003-0807-0

[47] Nakagawa, H. & Ishiwata, T. Effect of short- and long-term heat exposure on brain monoamines and emotional behavior in mice and rats. *J. Therm. Biol.* **99**, 102923 (2021). 34420602 10.1016/j.jtherbio.2021.102923

[48] Bajgiran, M., Azlan, A., Shamsuddin, S., Azzam, G. & Halim, M. A. Data on RNA-seq analysis of *Drosophila melanogaster* during ageing. *Data Brief.* **38**, 107413 (2021). 34632013 10.1016/j.dib.2021.107413 PMC8488473

[49] Wang, R. et al. Global stable-isotope tracing metabolomics reveals system-wide metabolic alternations in aging Drosophila. *Nat. Commun.* **13**, 3518 (2022). 35725845 10.1038/s41467-022-31268-6 PMC9209425

[50] Tower, J. Heat shock proteins and Drosophila aging. *Exp. Gerontol.* **46**, 355–362 (2011). 20840862 10.1016/j.exger.2010.09.002 PMC3018744

[51] Hwangbo, D. S., Gershman, B., Tu, M. P., Palmer, M. & Tatar, M. Drosophila dFOXO controls lifespan and regulates insulin signalling in brain and fat body. *Nature* **429**, 562–566 (2004). 15175753 10.1038/nature02549

[52] Rogers, R. P. & Rogina, B. The role of INDY in metabolism, health and longevity. *Front. Genet.* **6**, 204 (2015). 26106407 10.3389/fgene.2015.00204 PMC4460575

[53] Partridge, L., Piper, M. D. & Mair, W. Dietary restriction in Drosophila. *Mech. Ageing Dev.* **126**, 938–950 (2005). 15935441 10.1016/j.mad.2005.03.023

[54] Ma, Z. et al. Epigenetic drift of H3K27me3 in aging links glycolysis to healthy longevity in Drosophila. *Elife*10.7554/eLife.35368 (2018). 10.7554/eLife.35368 PMC5991832 29809154

[55] Issa, N. et al. The circulating protease persephone is an immune sensor for microbial proteolytic activities upstream of the Drosophila toll pathway. *Mol. Cell* **69**, 539–550.e536 (2018). 29452635 10.1016/j.molcel.2018.01.029 PMC5823974

[56] Takehana, A. et al. Peptidoglycan recognition protein (PGRP)-LE and PGRP-LC act synergistically in Drosophila immunity. *EMBO J.* **23**, 4690–4700 (2004). 15538387 10.1038/sj.emboj.7600466 PMC533052

[57] Shan, S. & Hoffman, J. M. Serine metabolism in aging and age-related diseases. *Geroscience* **47**, 611–630 (2025). 39585647 10.1007/s11357-024-01444-1 PMC11872823

[58] Lockwood, B. L., Julick, C. R. & Montooth, K. L. Maternal loading of a small heat shock protein increases embryo thermal tolerance in *Drosophila melanogaster*. *J. Exp. Biol.* **220**, 4492–4501 (2017). 29097593 10.1242/jeb.164848 PMC5769566

[59] Gong, W. J. & Golic, K. G. Loss of Hsp70 in Drosophila is pleiotropic, with effects on thermotolerance, recovery from heat shock and neurodegeneration. *Genetics* **172**, 275–286 (2006). 16204210 10.1534/genetics.105.048793 PMC1456155

[60] van der Straten, A., Rommel, C., Dickson, B. & Hafen, E. The heat shock protein 83 (Hsp83) is required for Raf-mediated signalling in Drosophila. *EMBO J.* **16**, 1961–1969 (1997). 9155022 10.1093/emboj/16.8.1961 PMC1169799

[61] Vos, M. J. et al. Specific protein homeostatic functions of small heat-shock proteins increase lifespan. *Aging Cell* **15**, 217–226 (2016). 26705243 10.1111/acel.12422 PMC4783350

[62] de Magalhaes, J. P. et al. Human Ageing Genomic Resources: updates on key databases in ageing research. *Nucleic Acids Res.* **52**, D900–D908 (2024). 37933854 10.1093/nar/gkad927 PMC10767973

[63] Da Lage, J. L., Renard, E., Chartois, F., Lemeunier, F. & Cariou, M. L. Amyrel, a paralogous gene of the amylase gene family in *Drosophila melanogaster* and the Sophophora subgenus. *Proc. Natl. Acad. Sci. USA* **95**, 6848–6853 (1998). 9618501 10.1073/pnas.95.12.6848 PMC22658

[64] HATTORI, K. et al. Cloning, expression, and characterization of cytosolic sulfotransferase isozymes from *Drosophila melanogaster*. *Biosci., Biotechnol., Biochem.* **72**, 540–547 (2008). 18256476 10.1271/bbb.70647

[65] Chung, H. et al. The Drosophila homolog of methionine sulfoxide reductase A extends lifespan and increases nuclear localization of FOXO. *FEBS Lett.* **584**, 3609–3614 (2010). 20655917 10.1016/j.febslet.2010.07.033

[66] Lepesant, J. A. et al. Developmentally regulated gene expression in Drosophila larval fat bodies. *J. Mol. Appl. Genet.* **1**, 371–383 (1982). 6818315

[67] Miller, H. A. et al. Serotonin and dopamine modulate aging in response to food odor and availability. *Nat. Commun.* **13**, 3271 (2022). 35672307 10.1038/s41467-022-30869-5 PMC9174215

[68] Rahman, S. S. et al. Methionine cycle in *C. elegans* serotonergic neurons regulates diet-dependent behaviour and longevity through neuron-gut signaling. *Nat. Commun.* **16**, 5118 (2025). 40456752 10.1038/s41467-025-60475-0 PMC12130505

[69] Rosikon, K. D., Bone, M. C. & Lawal, H. O. Regulation and modulation of biogenic amine neurotransmission in Drosophila and Caenorhabditis elegans. *Front Physiol.* **14**, 970405 (2023). 36875033 10.3389/fphys.2023.970405 PMC9978017

[70] Kebbede, L., Weathers, B. & Nair, T. The connection between serotonin signaling, aging, and immunity when observed from *C. Elegans* and human studies. *Innov. Aging* **8**, 1166–1167 (2024).

[71] Reches, A. et al. Serotonin depletion induced by reserpine is attenuated by prophylactic administration of lithium. *Eur. J. Pharm.* **113**, 225–231 (1985). 10.1016/0014-2999(85)90739-3 2412842

[72] Adamo, S. A., Linn, C. E. & Hoy, R. R. The role of neurohormonal octopamine during ‘fight or flight’ behaviour in the field cricket *Gryllus bimaculatus*. *J. Exp. Biol.* **198**, 1691–1700 (1995). 7636443 10.1242/jeb.198.8.1691

[73] Roeder, T. TYRAMINE AND OCTOPAMINE: ruling behavior and metabolism. *Annu. Rev. Entomol.* **50**, 447–477 (2005). 15355245 10.1146/annurev.ento.50.071803.130404

[74] Cichewicz, K. et al. A new brain dopamine-deficient Drosophila and its pharmacological and genetic rescue. *Genes Brain Behav.* **16**, 394–403 (2017). 27762066 10.1111/gbb.12353 PMC5492937

[75] Simon, A. F. et al. Drosophila vesicular monoamine transporter mutants can adapt to reduced or eliminated vesicular stores of dopamine and serotonin. *Genetics* **181**, 525–541 (2009). 19033154 10.1534/genetics.108.094110 PMC2644945

[76] Franceschi, C. & Campisi, J. Chronic inflammation (inflammaging) and its potential contribution to age-associated diseases. *J. Gerontol. A Biol. Sci. Med. Sci.* **69**, S4–S9 (2014). 24833586 10.1093/gerona/glu057

[77] Papadopoli, D. et al. mTOR as a central regulator of lifespan and aging. *F1000Res*10.12688/f1000research.17196.1 (2019). 10.12688/f1000research.17196.1 PMC6611156 31316753

[78] Blagosklonny, M. V. Calorie restriction: decelerating mTOR-driven aging from cells to organisms (including humans). *Cell Cycle* **9**, 683–688 (2010). 20139716 10.4161/cc.9.4.10766

[79] Redman, L. M. et al. Metabolic slowing and reduced oxidative damage with sustained caloric restriction support the rate of living and oxidative damage theories of aging. *Cell Metab.* **27**, 805–815.e804 (2018). 29576535 10.1016/j.cmet.2018.02.019 PMC5886711

[80] Singhal, S. S. et al. Antioxidant role of glutathione S-transferases: 4-Hydroxynonenal, a key molecule in stress-mediated signaling. *Toxicol. Appl. Pharm.* **289**, 361–370 (2015). 10.1016/j.taap.2015.10.006 PMC4852854 26476300

[81] Finkel, T. The metabolic regulation of aging. *Nat. Med.* **21**, 1416–1423 (2015). 26646498 10.1038/nm.3998

[82] Katewa, S. D. et al. Intramyocellular fatty-acid metabolism plays a critical role in mediating responses to dietary restriction in *Drosophila melanogaster*. *Cell Metab.* **16**, 97–103 (2012). 22768842 10.1016/j.cmet.2012.06.005 PMC3400463

[83] Zur Lage, P., Newton, F. G. & Jarman, A. P. Survey of the ciliary motility machinery of Drosophila sperm and ciliated mechanosensory neurons reveals unexpected cell-type specific variations: a model for motile ciliopathies. *Front. Genet.* **10**, 24 (2019). 30774648 10.3389/fgene.2019.00024 PMC6367277

[84] Rogers, R. P. & Rogina, B. Increased mitochondrial biogenesis preserves intestinal stem cell homeostasis and contributes to longevity in Indy mutant flies. *Aging* **6**, 335–350 (2014). 24827528 10.18632/aging.100658 PMC4032799

[85] Beifuss, M. J. & Durica, D. S. Sequence analysis of the indirect flight muscle actin-encoding gene of Drosophila simulans. *Gene* **118**, 163–170 (1992). 1511890 10.1016/0378-1119(92)90185-r

[86] Dohn, T. E. & Cripps, R. M. Absence of the Drosophila jump muscle actin Act79B is compensated by up-regulation of Act88F. *Dev. Dyn.* **247**, 642–649 (2018). 29318731 10.1002/dvdy.24616 PMC6118211

[87] Chechenova, M. B., Maes, S. & Cripps, R. M. Expression of the troponin C at 41C gene in adult Drosophila tubular muscles depends upon both positive and negative regulatory inputs. *PLoS ONE* **10**, e0144615 (2015). 26641463 10.1371/journal.pone.0144615 PMC4671713

[88] Agip, A. A., Chung, I., Sanchez-Martinez, A., Whitworth, A. J. & Hirst, J. Cryo-EM structures of mitochondrial respiratory complex I from *Drosophila melanogaster*. *Elife* **12**, e84424 (2023). 36622099 10.7554/eLife.84424 PMC9977279

[89] Rodriguez-Nuevo, A. et al. Oocytes maintain ROS-free mitochondrial metabolism by suppressing complex I. *Nature* **607**, 756–761 (2022). 35859172 10.1038/s41586-022-04979-5 PMC9329100

[90] Neckameyer, W. S. & White, K. Drosophila tyrosine hydroxylase is encoded by the pale locus. *J. Neurogenet.* **8**, 189–199 (1993). 8100577 10.3109/01677069309083448

[91] Daubner, S. C., Le, T. & Wang, S. Tyrosine hydroxylase and regulation of dopamine synthesis. *Arch. Biochem. Biophys.* **508**, 1–12 (2011). 21176768 10.1016/j.abb.2010.12.017 PMC3065393

[92] Huang, J., Liu, W., Qi, Y. X., Luo, J. & Montell, C. Neuromodulation of courtship drive through tyramine-responsive neurons in the Drosophila brain. *Curr. Biol.* **26**, 2246–2256 (2016). 27498566 10.1016/j.cub.2016.06.061 PMC5021585

[93] Duerr, J. S. et al. The *cat-1* gene of *Caenorhabditis elegans* encodes a vesicular monoamine transporter required for specific monoamine-dependent behaviors. *J. Neurosci.* **19**, 72–84 (1999). 9870940 10.1523/JNEUROSCI.19-01-00072.1999 PMC6782383

[94] Jiang, Y., Gaur, U., Cao, Z., Hou, S. T. & Zheng, W. Dopamine D1- and D2-like receptors oppositely regulate lifespan via a dietary restriction mechanism in Caenorhabditis elegans. *BMC Biol.* **20**, 71 (2022). 35317792 10.1186/s12915-022-01272-9 PMC8941781

[95] White, K. E., Humphrey, D. M. & Hirth, F. The dopaminergic system in the aging brain of Drosophila. *Front. Neurosci.* **4**, 205 (2010). 21165178 10.3389/fnins.2010.00205 PMC3002484

[96] Giacobbo, B. L. et al. The aged striatum: evidence of molecular and structural changes using a longitudinal multimodal approach in mice. *Front Aging Neurosci.* **14**, 795132 (2022). 35140600 10.3389/fnagi.2022.795132 PMC8818755

[97] Dekker, A. D. et al. Aging rather than aneuploidy affects monoamine neurotransmitters in brain regions of Down syndrome mouse models. *Neurobiol. Dis.* **105**, 235–244 (2017). 28624415 10.1016/j.nbd.2017.06.007 PMC5536154

[98] Li, X., Bäckman, L. & Persson, J. The relationship of age and DRD2 polymorphisms to frontostriatal brain activity and working memory performance. *Neurobiol. Aging* **84**, 189–199 (2019). 31629117 10.1016/j.neurobiolaging.2019.08.022

[99] Zheng, H.-Z. et al. High-throughput measurement of *Drosophila* feeding behavior. *Cell Rep. Methods*10.1016/j.crmeth.2025.101109 (2025). 10.1016/j.crmeth.2025.101109 PMC12461584 40683246

[100] Soukas, A. A., Hao, H. & Wu, L. Metformin as anti-aging therapy: is it for everyone?*Trends Endocrinol. Metab.* **30**, 745–755 (2019). 31405774 10.1016/j.tem.2019.07.015 PMC6779524

[101] Pillarisetti, L. & Agrawal, D. K. Semaglutide: double-edged sword with risks and benefits. *Arch. Intern. Med. Res.* **8**, 1–13 (2025). 39902055 10.26502/aimr.0189 PMC11790292

[102] Grotewiel, M. S., Martin, I., Bhandari, P. & Cook-Wiens, E. Functional senescence in *Drosophila melanogaster*. *Ageing Res. Rev.* **4**, 372–397 (2005). 16024299 10.1016/j.arr.2005.04.001

