# Supplementary material for: Reserpine prolongs lifespan but compromises locomotion and heat-stress resilience in Drosophila melanogaster
Source: NPJ Aging. 2026 Jan 12;12(1):21. doi: 10.1038/s41514-026-00329-1 (PMC12858949; doi:10.1038/s41514-026-00329-1)
Supplement: Supplementary file 1 — Supplementary Information [file 41514_2026_329_MOESM1_ESM.pdf]

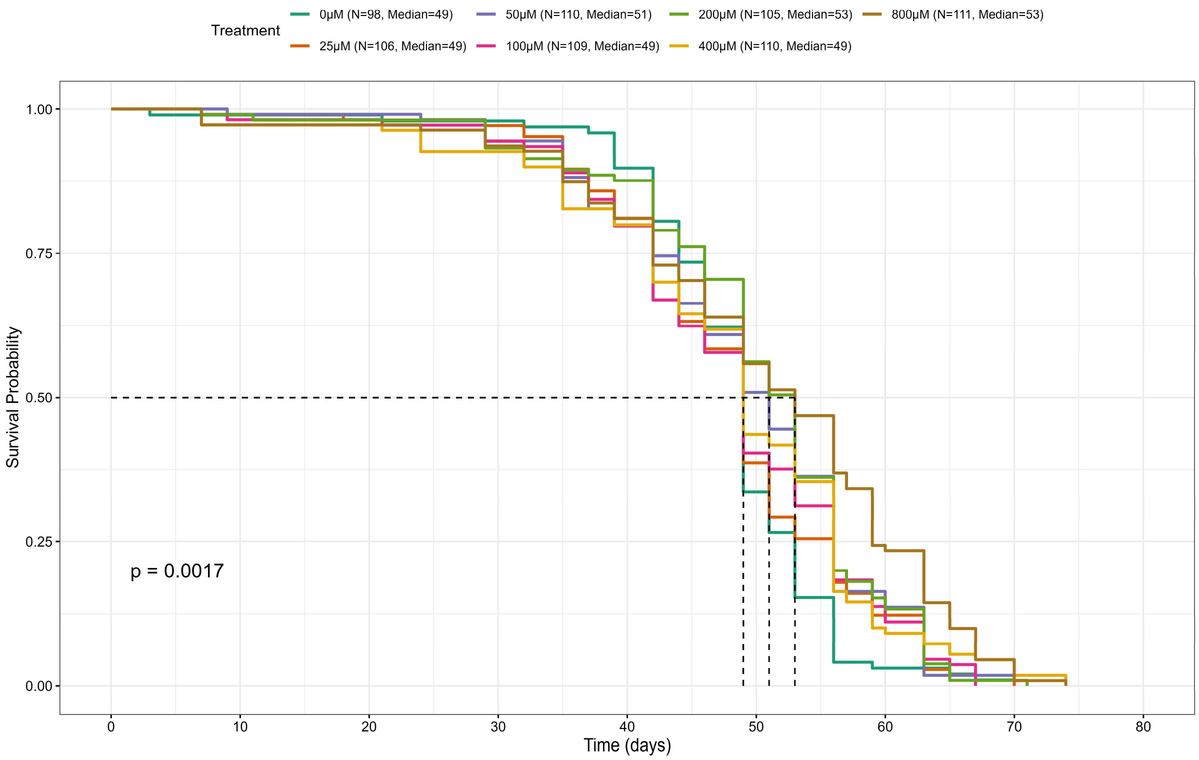

Figure S1. Pilot study assessing median survival at lower reserpine concentrations.

Kaplan–Meier survival curves for male flies chronically treated with (0, 25, 50, 100, 200, 400, and 800 µM). Median survival values were: 49 (0 µM), 49 (25 µM), 51 (50 µM), 49 (100 µM), 53 (200 µM), 49 (400 µM), and 53 (800 µM). Each treatment group included 3 independent biological replicates each containing an average of n ~ 35 flies per vial. Overall group differences were significant (log-rank test, $p < 0.0017$).

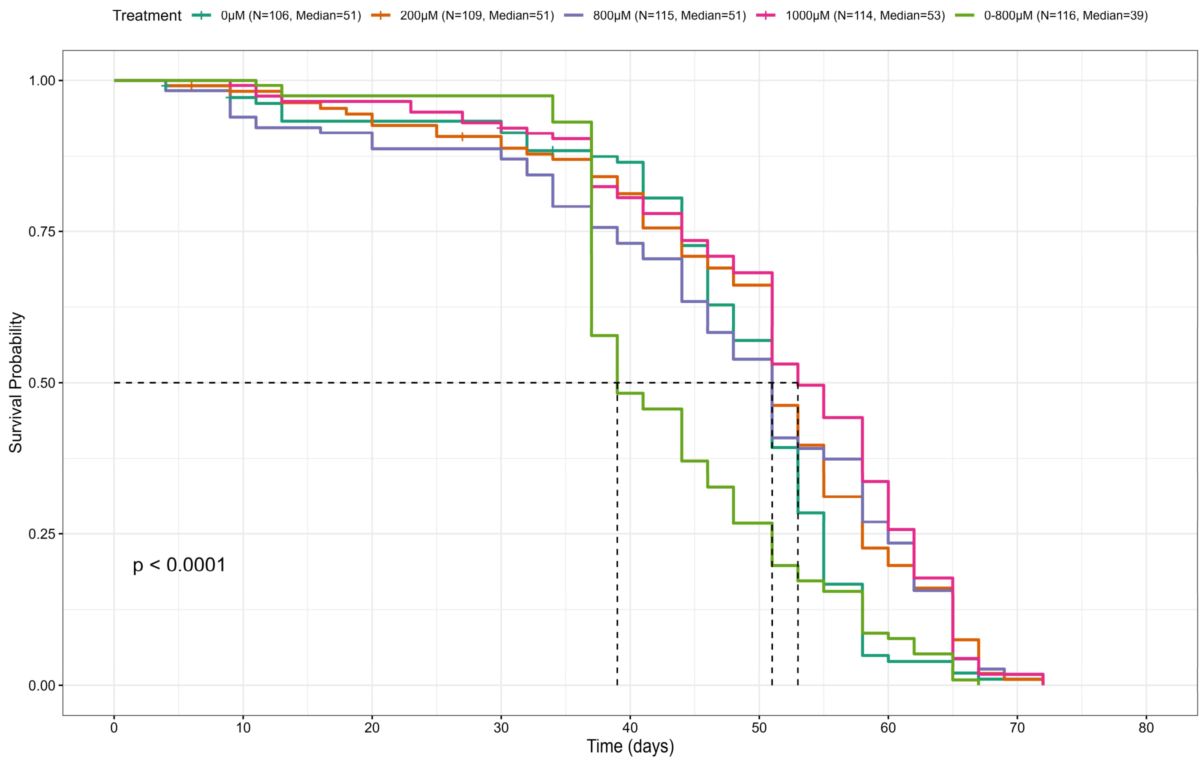

Figure S2. Follow-up pilot study showing survival at selected concentrations and a late-life intervention group.

Kaplan–Meier survival curves for male flies chronically treated with Median survival values were: 51 (0 µM), 51 (200 µM), 51 (800 µM), 55 (1000 µM), and 39 (0-800 µM; reserpine introduced at day 31). These data suggest that timing of exposure when young is critical for the beneficial effects of reserpine. Each treatment group included 3 independent biological replicates each containing an average of n ~ 35 flies per vial. Overall group differences were significant (log-rank test, $p < 0.0001$).

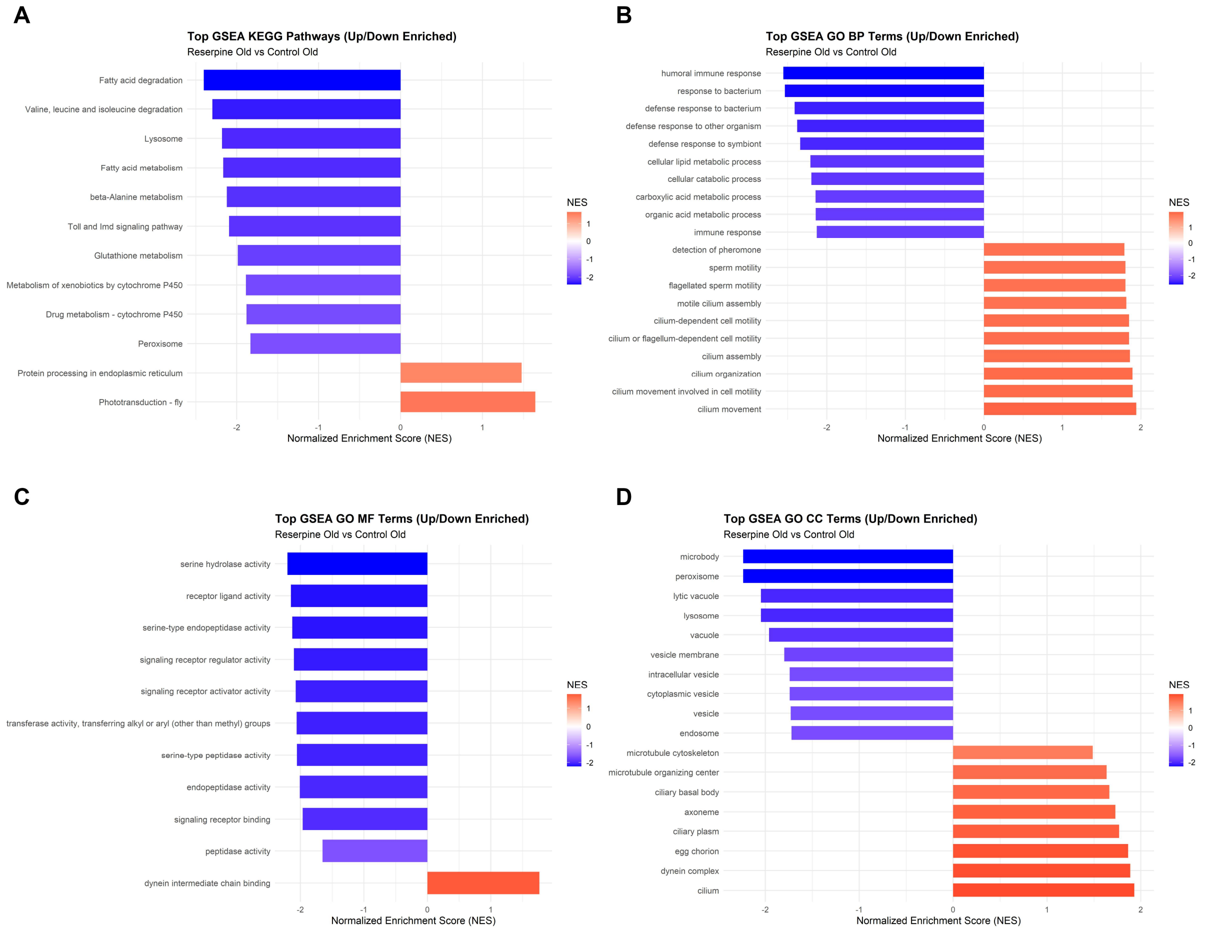

Figure S3. Gene Set Enrichment Analysis in Old Confirm global downregulation of immune and stress response pathways. (Whole gene set is used in GSEA pAdjustMethod = "BH", pvalueCutoff = 0.05, minGSSize = 10, maxGSSize = 500.)

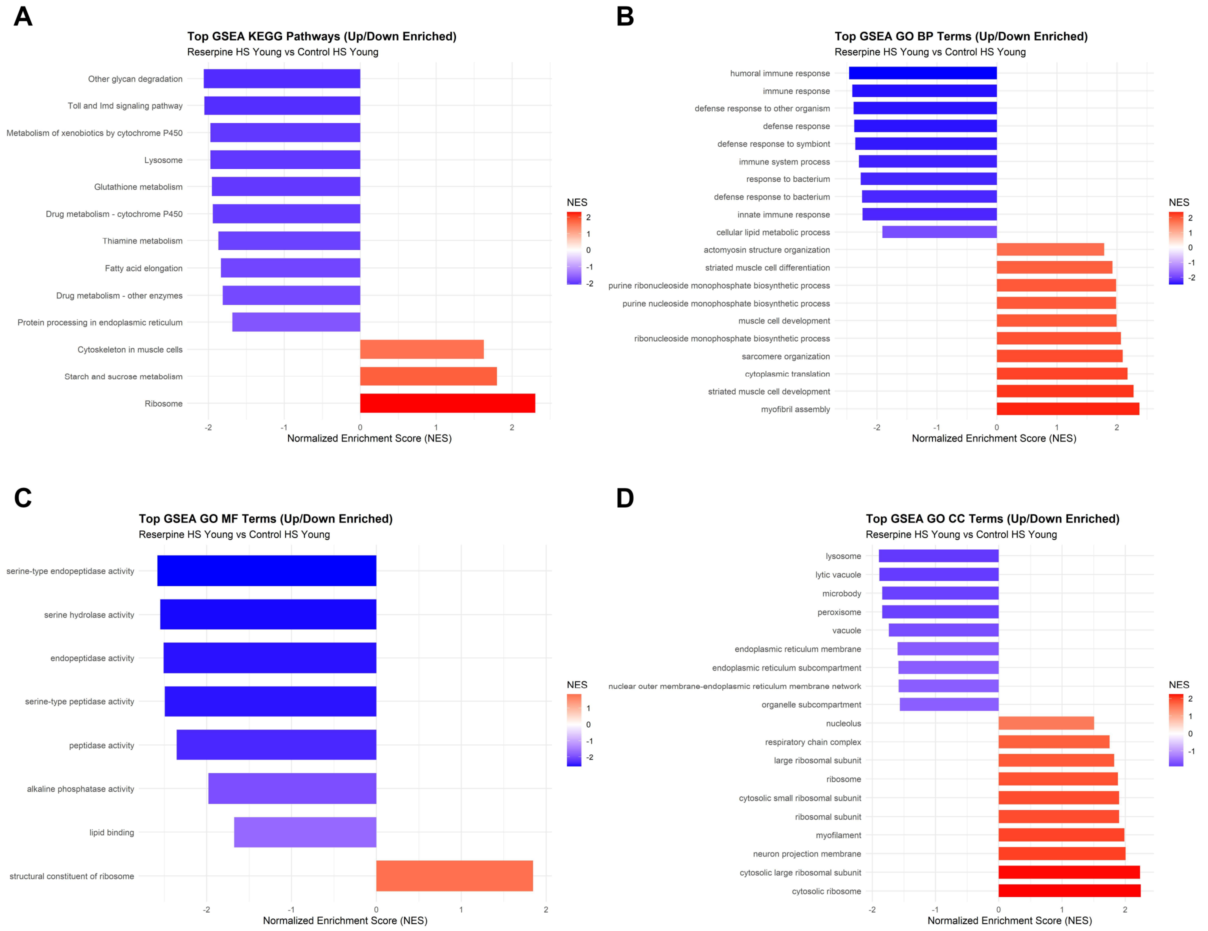

Figure S4. Gene Set Enrichment Analysis in Young Confirm global downregulation of immune and stress response pathways from early age. (Whole gene set is used in GSEA pAdjustMethod = "BH", pvalueCutoff = 0.05, minGSSize = 10, maxGSSize = 500.)

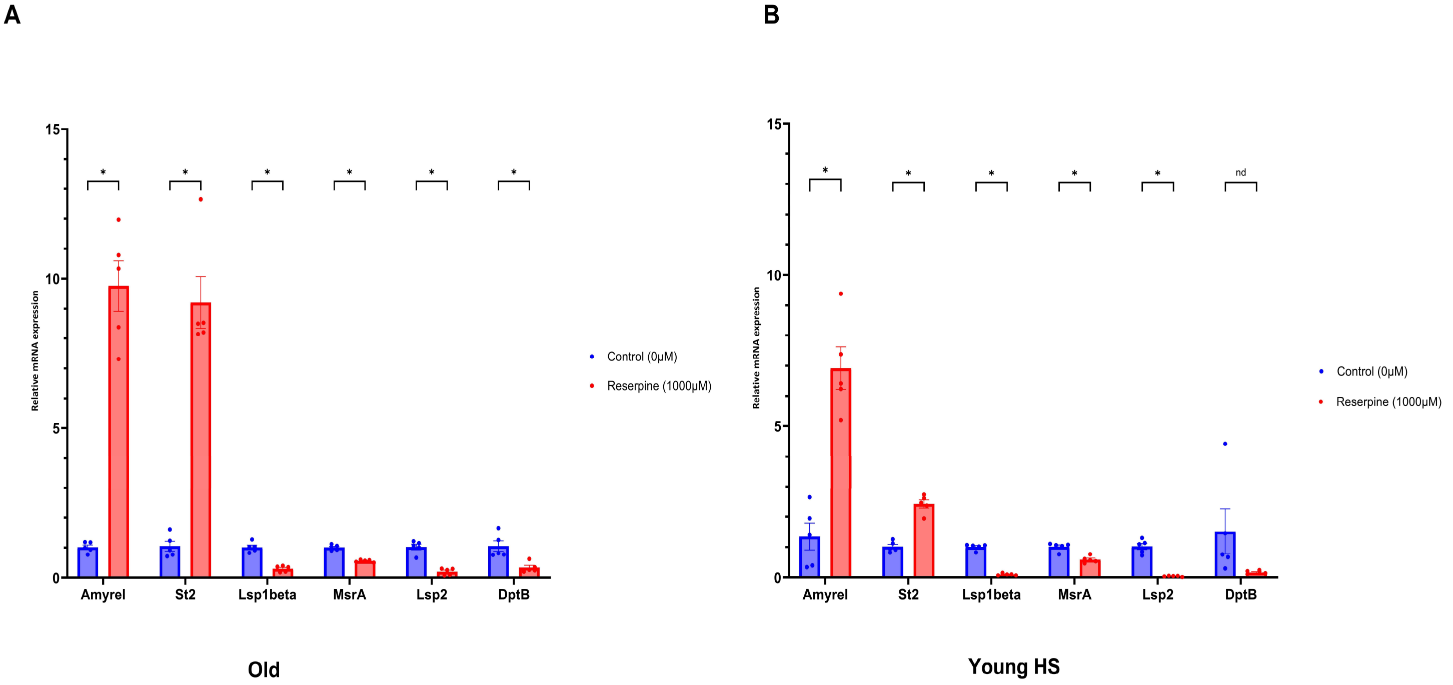

Figure S5: RT-qPCR validation of RNA-seq targets in Old and Heat-Stressed Young flies. (A) Relative mRNA expression of selected target genes in aged flies (Old) treated with 0 μM (Control) or 1000 μM Reserpine. Reserpine treatment significantly upregulates *Amyrel* and *St2* while downregulating genes involved in stress response and storage (*Lsp1beta, MsrA, Lsp2, DbtB*). (B) Relative mRNA expression in young flies subjected to Heat Stress (Young HS). Data are presented as mean ± S.E.M. Individual dots represent biological replicates (n = 5 per group). Statistical significance was determined using multiple unpaired *t*-tests with False Discovery Rate (FDR) correction (Two-stage Benjamini, Krieger, and Yekutieli method; $q < 0.05$).

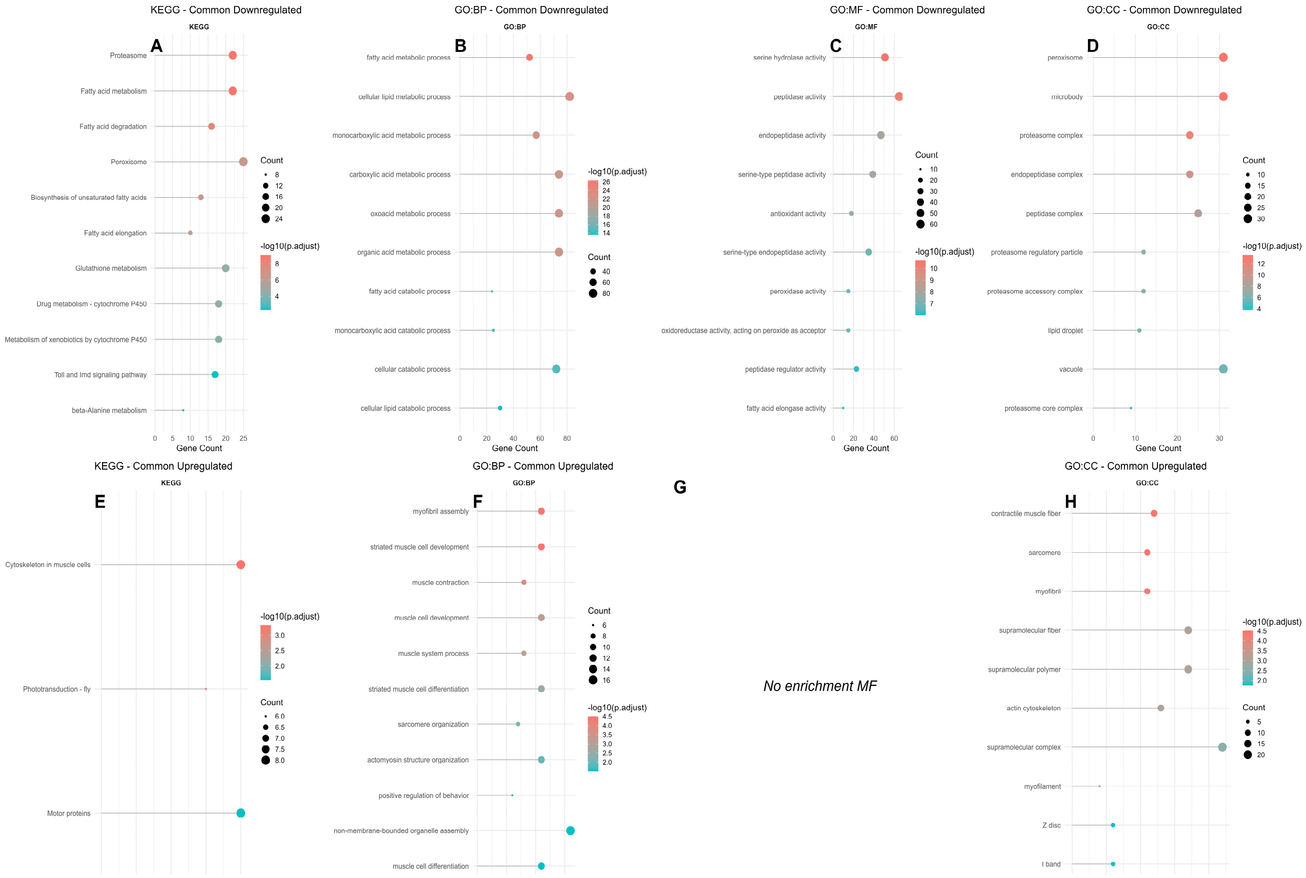

Figure S6. Comparison of transcriptomic changes between aging and heat shock reserpine vs control flies.

(Enrichment of Overlapping downregulated DEGs (pvalueCutoff = 0.05, minGSSize =10, maxGSSize = 500) Enrichment of Overlapping Upregulated DEGs (pvalueCutoff = 0.05, minGSSize = 10, maxGSSize = 500)

Gene Symbol	FlyBase Gene ID	Functional Category
**ple**	FBgn0005626	Tyrosine hydroxylase (dopamine synthesis)
**Punch**	FBgn0003162	GTP cyclohydrolase I (cofactor biosynthesis)
**Ddc**	FBgn0000422	Dopa decarboxylase (dopamine/serotonin synthesis)
**Tph**	FBgn0262139	Tryptophan hydroxylase (serotonin synthesis)
**Tdc2**	FBgn0050446	Tyrosine decarboxylase (tyramine/octopamine synthesis)
**Vmat**	FBgn0260964	Vesicular monoamine transporter
**DAT**	FBgn0034136	Dopamine transporter
**SerT**	FBgn0010414	Serotonin transporter
**Ebony**	FBgn0000527	Dopamine metabolism (β-alanyl-dopamine synthesis)
**CG10433**	FBgn0034638	Predicted monoamine transporter
**Dop2R**	FBgn0053517	Dopamine receptor 2
**5-HT1A**	FBgn0004168	Serotonin receptor 1A
**5-HT1B**	FBgn0263116	Serotonin receptor 1B
**5-HT2A**	FBgn0087012	Serotonin receptor 2A
**5-HT2B**	FBgn0261929	Serotonin receptor 2B
**5-HT7**	FBgn0004573	Serotonin receptor 7
**Oct-TyrR**	FBgn0004514	Octopamine/tyramine receptor
**Oamb**	FBgn0024944	Octopamine receptor in mushroom bodies
**TyrR**	FBgn0038542	Tyramine receptor
**norpA**	FBgn0262738	Phospholipase C (neurotransmission)
**nSyb**	FBgn0013342	Synaptic vesicle protein
**CSP**	FBgn0004179	Synaptic chaperone (Cysteine String Protein)
**Shaker**	FBgn0003380	Voltage-gated potassium channel

**para**	FBgn0285944	Voltage-gated sodium channel
**cac**	FBgn0263111	Voltage-gated calcium channel
**ATPsyn-beta**	FBgn0010217	ATP synthase β subunit (mitochondrial)
**ND-75**	FBgn0017566	NADH dehydrogenase subunit 75 kDa
**SdhA**	FBgn0000181	Succinate dehydrogenase subunit A
**COX5A**	FBgn0019624	Cytochrome c oxidase subunit 5A
**Mhc**	FBgn0264695	Myosin heavy chain (muscle function)
**Act88F**	FBgn0000047	Actin 88F (muscle-specific actin)
**TpnC41C**	FBgn0013348	Troponin C isoform (muscle)
**Tm1**	FBgn0003721	Tropomyosin 1 (muscle)
**per**	FBgn0003068	Period (circadian rhythm)
**tim**	FBgn0014396	Timeless (circadian rhythm)
**clk**	FBgn0023076	Clock (circadian rhythm)
**cyc**	FBgn0023091	Cycle (circadian rhythm)
**Pdf**	FBgn0015271	Pigment dispersing factor (circadian output)
**mt: ND3**	FBgn0013681	mitochondrial NADH-ubiquinone oxidoreductase chain 3

Table S1: List of gene tested, related to monoamine depletion used for comparison to the mRNA seq data.

Gene Symbol	FBgn ID	Log2FC (YoungHS)	Log2FC (Old)	Regulation Pattern in mRNA seq
**Amyrel**	FBgn0020506	1,95	2,87	Up in Both
**St2**	FBgn0037665	1,16	2,86	Up in Both
**Lsp2**	FBgn0002565	-4,28	-2,14	Down in Both
**MsrA**	FBgn0000565	-0,86	-1	Down in Both
**Lsp1β**	FBgn0002563	-3,1	-1,57	Down in Both
**DptB**	FBgn0034407	-3,14	-1,46	Down in Both

Table S2 : Genes selected for qRT-PCR validation from mRNA seq data.

Gene	Primer	Sequence	Annealing temp
Amyrel	Dm-Amyrel- fw1	GGCATCGGAAGATCTAGAGTACAT	60
	Dm-Amyrel- rv2	AGGGCAAGAATCCCCAATCG	
St2	Dm-St2-fw1	GAGACGGTGGCCCAAAAGT	60
	Dm-St2-rv1	GAGACTTTGAACGCCCAGGA	
Lsp1beta	Dm-Lsp1beta-fw1	GGAGTCCTACTACACTCACTACG	60
	Dm-Lsp1beta-rv1	AGTCGAACTTCTCCGCTTCG	
MsrA	Dm-MsrA-fw2	TTCGGCATGGGTTGCTTTTG	60
	Dm-MsrA-rv2	CCTCCGTATGATCGCCCATTT	
Lsp2	Dm-Lsp2-fw1	TGTACACCTTCGGCAGGAAC	60
	Dm-Lsp2-rv1	TAGTCACTGTTGGTGGCGTC	
DptB	Dm-DptB-fw1	GTGCCTGGGCTTATCCCTATC	60
	Dm-DptB-rv1	AAATTGGGAGCATATGCCAGTG	
RPL32 (ref gene)	Rpl32CG7939-fw	CCAGTCGGATCGATATGCTA	60
	Rpl32CG7939-rv	GTTCGATCCGTAACCGATGT	
Gapdh2 (ref gene)	Gapdh2CG8893-fw	CGTTCATGCCACCACCGCTA	60
	Gapdh2CG8893-RV	CCACGTCCATCACGCCACAA	

Table S3 : Primers selected for qRT-PCR validation.
